# Single-Nucleus Transcriptomic Sequencing Revealed Cellular and Molecular Changes in a Pilocarpine-Induced Epilepsy Rat Model

**DOI:** 10.1007/s12264-025-01451-y

**Published:** 2025-07-24

**Authors:** Ying Wang, Yue Wang, Fei Yu, Yidi Liu, Xin Liu, Zhengxu Cai

**Affiliations:** 1 Department of Neurology, The First Affiliated Hospital of Dalian Medical University, Dalian, 116011 China; 2 State Key Laboratory of Quality Research in Chinese Medicine and Institute of Chinese Medical Sciences, University of Macau, Macao, 51900 China; 3 Shanxi Medical University, Taiyuan, 030607 China; 4 University of Chinese Academy of Sciences, Beijing, 100049 China

**Keywords:** Epileptogenesis, Single-nucleus RNA sequencing, Pilocarpine-induced epilepsy rat model, Latent phase, Astrocytes

## Abstract

Uncovering the underlying process of epileptogenesis is crucial for developing effective treatment strategies for epilepsy. However, the cellular and molecular changes throughout epileptogenesis are not fully understood. In this study, single-nucleus RNA sequencing was performed on the hippocampus, temporal cortex, and thalamus across the acute, latent, and chronic phases in a pilocarpine-induced rat model and controls. We created a comprehensive single-nucleus transcriptomic atlas of rat epileptogenesis, consisting of 311,177 single nuclei. Our analysis revealed distinct transcriptional signatures across the three phases and regions, including significant gene expression changes in the acute phase and critical synaptic and neural network remodeling in the thalamus during the latent phase. Notably, we identified two novel astrocyte clusters during epileptogenesis, with the EX-Astro C3-IN pathway emerging as a potential intervention target. The dataset provides a detailed understanding of the dynamic cellular and molecular landscape of epileptogenesis.

**Supplementary Information:**

The online version contains supplementary material available at 10.1007/s12264-025-01451-y.

## Introduction

Epilepsy, a chronic brain disorder characterized by spontaneous recurrent seizures (SRSs) with cognitive and psychological comorbidities, affects approximately 1% of the global population and ranks as the third most common neurological condition [1]. The pathophysiology of epilepsy is complex, and antiseizure medications (ASMs) primarily target symptomatic seizure control. Despite the development of new ASMs over the past 30 years, approximately 30% of epilepsy patients fail to achieve complete remission [2–4]. This limitation is partly due to the inability of ASMs to prevent the onset and progression of epilepsy [5]. Therefore, understanding the cellular and molecular mechanisms underlying epileptogenesis is critical for identifying new therapeutic targets.

Epileptogenesis refers to the process by which a normal brain, under certain genetic or acquired influences, transforms into an epileptic brain exhibiting SRSs [6, 7]. This process is considered to be divided into three key phases: the acute phase, involving transient brain insults such as trauma, infection, stroke, tumor, or status epilepticus (SE), typically lasting for the first 3 d following SE in epilepsy models [8, 9]. The latent phase is a silent period without ictal seizures. Research suggests that the latent period of the pilocarpine (PILO)-induced epilepsy model ranges from one to six weeks, with an average duration of approximately two weeks [10, 11]. The chronic phase is characterized by the onset of SRSs, indicating a complete transition to an epileptic brain [6]. To ensure accurate tissue sampling for this study, we designated 24 h, 7 d, and 8 w post-SE as representative time points for the acute, latent, and chronic phases, respectively.

Temporal lobe epilepsy (TLE), typically originating from the limbic system, including the hippocampus and amygdala, is the most common type of focal epilepsy and the most prevalent refractory epilepsy in adults [12]. However, studying epileptogenesis in human TLE tissue poses significant challenges. These include a limited supply of nonepileptic, age-matched human control brain tissue samples and the fact that surgical samples are often obtained from advanced epilepsy stages rather than early epileptogenesis [13], introducing confounding factors into analyses. The PILO rodent model serves as a classical and clinically relevant model of TLE, with pathological, electrophysiological, and seizure patterns closely resembling those of human TLE. While the hippocampus has been predominantly studied in TLE, dysregulation across other brain regions and neural networks also plays a crucial role in epileptogenesis [14, 15]. For instance, a previous study demonstrated activation of epileptic networks in both the cortex and hippocampus in response to visual or auditory stimuli in kainic acid TLE mice [16]. The thalamus, as a central hub of cortical-thalamocortical circuits, integrates excitatory projections and transmits inputs to the cerebral cortex through diverse anatomical connectivity patterns [17]. It drives seizure propagation in generalized epilepsies (e.g., absence seizures) and influences secondary generalization in focal epilepsies such as TLE [18]. Moreover, the thalamus has been used as a neurostimulation target for the treatment of epilepsy in clinical practice [18–20]. Despite its importance, the precise role of the thalamus in epileptogenesis remains unclear. Additionally, hippocampal degeneration, commonly observed in TLE patients, complicates the collection of comparative data [21]. Therefore, this study included the hippocampus, temporal cortex, and thalamus to comprehensively investigate the mechanisms of epileptogenesis.

Recently, single-cell RNA sequencing (scRNA-Seq) and single-nucleus RNA sequencing (snRNA-Seq) have provided insights into how various cell populations contribute to epilepsy [21–23]. However, changes at the single-cell level throughout epileptogenesis remain poorly understood. This study aimed to investigate the temporal (acute, latent, and chronic phases) and spatial (hippocampal, temporal cortex, and thalamic) aspects of epileptogenesis in a PILO rat model of TLE using snRNA-Seq analysis.

## Materials and Methods

### Animals

The study was conducted using 6- to 8-week-old male Sprague Dawley rats. The environment was carefully controlled, maintaining a temperature of 22 ± 2°C, humidity at 50% ± 5%, and a 12-hour light/dark cycle. Food and water were provided ad libitum, and the rats were allowed to adapt to the environment for one week prior to the experiment. All procedures adhered to the guidelines for the care and use of laboratory animals. The experimental protocol was approved by the Animal Ethics Committee of Dalian Medical University (file number AEE22073)

### Lithium-PILO Rat Model of TLE

The lithium-PILO rat model of TLE was employed in this study [11, 14]. Briefly, rats received an intraperitoneal (i.p.) injection of lithium chloride (127 mg/kg; Sigma-Aldrich, USA) 19–20 h prior to PILO administration. To inhibit peripheral muscarinic receptors, methylscopolamine nitrate (1 mg/kg; i.p., Macklin, China) was administered 30 min before the PILO injection. Subsequently, PILO was given (initial dose: 20 mg/kg; i.p., Sigma-Aldrich, USA, followed by 10 mg/kg every 30 min until SE) to induce SE. Seizures were assessed using the Racine scale [24], with grade 4 or 5 seizures lasting at least 30 min and failing to resolve identified as SE. To terminate convulsions, diazepam (10 mg/kg; i.p., Tianjin, China) was administered one hour after SE onset. The control group underwent the same procedure, with saline replacing PILO.

### Behavior Monitoring

Continuous 24-hour video monitoring was conducted on the experimental animals during the first seven days post-modeling to confirm that none of the animals in the latent phase exhibited spontaneous seizures before sampling. From the eighth day onward, monitoring was performed daily from 6:00 to 18:00 (the light cycle for the animals) over a span of seven weeks. Only seizures of Racine scale grade 4 or higher, lasting more than 10 s, were considered spontaneous seizures. The interval between two SRSs was required to be at least one hour; otherwise, they were counted as a single seizure. The latency period (time from SE to the first spontaneous seizure), as well as the number and duration of SRSs in rats that survived and developed SRSs, were documented. Rats that did not experience SRSs during the eight-week observation period were excluded from the study.

### Electroencephalography (EEG) Recordings

SRSs were verified via EEG recordings performed on a subset of animals from the same experimental batch used for snRNA-Seq to confirm the successful establishment of the PILO-induced epilepsy rat model, as electrode implantation can influence gene expression in the brain. EEG recordings were conducted in the fourth week after modeling in both the control and chronic groups. Rats were anesthetized with isoflurane (RWD Life Science, China) and secured in a stereotaxic frame (RWD Life Science, China). Three stainless steel screws (1 mm diameter, 3 mm length) were implanted at an AP of −3.0 mm and an ML of +/−4 mm as the recording and reference electrodes, respectively. The ground electrode was positioned at an AP of −6.0 mm and an ML of +2.5 mm. These screws were connected to a custom terminal and affixed to the skull using dental cement. After surgery, the rats were allowed a three-day recovery period. Subsequently, the electrode terminal was connected to the EEG recording system (Nihon Kohden EEG 1200-C system, Japan), and EEG signals were recorded for four hours during daylight. Spontaneous seizures were identified as evolving spike-wave discharges with a frequency >2 Hz and an amplitude three times the baseline, lasting for at least 15 s [25, 26].

### Tissue Preparation

The designated time points corresponding to the acute, latent, and chronic phases of epileptogenesis were 24 h, 7 d, and 8 w post-SE, respectively. A control group was included, which received a saline injection as a substitute for PILO after 7 d. At these time points, the rats were euthanized via an overdose of isoflurane (RWD Life Science, China) inhalation. The hippocampus, temporal cortex, and thalamus from both brain hemispheres were carefully dissected and stored at -80°C for subsequent RNA extraction for qRT-PCR and snRNA-seq. For histological analysis, the rats were deeply anesthetized using 5% isoflurane (RWD Life Science, China) inhalation and transcardially perfused with 250–300 mL of cold phosphate-buffered saline (PBS) to remove blood from the vasculature. The bilateral brain hemispheres were collected and preserved in 4% paraformaldehyde (Biosharp, China). All sample collections were performed before noon to minimize circadian influences on gene expression.

### Nissl Staining

Rat brains were fixed in 4% paraformaldehyde, dehydrated, embedded in paraffin, and sectioned into 4 μm-thick coronal slices. The sections were dewaxed, stained with toluidine blue (Servicebio, China) for 2–5 min, and rinsed with distilled water for 5 min. They were then slightly differentiated using 0.1% glacial acetic acid, with the reaction terminated by washing under running water. The samples were subsequently dried in an oven, cleared with xylene for 10 min, and sealed with neutral gum.

### RNA Extraction and Quantitative Real-Time PCR (qRT-PCR)

Total RNA from the hippocampus was extracted using the RNAsimple Total RNA Kit (TIANGEN, China) according to the manufacturer’s instructions. cDNA was synthesized using the PrimeScript^TM^ reagent kit (TaKaRa, China). qRT-PCR was conducted on a QuantStudio Dx Real-Time PCR System (Applied Biosystems) with the TB Green Premix Ex Taq^TM^ II kit (TaKaRa, China). The primers used are listed in Table 1. Relative gene expression levels were normalized to *Gapdh* and calculated using the 2^-ΔΔCt^ method.

**Table 1:** Primers used in this study.

Genes	Primer sequences (5’-3’)
Gapdh F	GGTCATCAACGGGAAACCCA
Gapdh R	CGACATACTCAGCACCAGCA
C1qa F	GGGCTCTTCCAGGTGTTAGC
C1qa R	CTGGTAAATGCGGCCCTTTG
C3 F	ATCGAGGATGGTTCAGGGGA
C3 R	GCCTCTACCATGTCGCTACC
Il-1β F	CCTATGTCTTGCCCGTGGAG
Il-1β R	CACACACTAGCAGGTCGTCA
Tnf-α F	ATGGGCTCCCTCTCATCAGT
Tnf-α R	GCTTGGTGGTTTGCTACGAC
vGlut1 F	GAGTCACCTGCACTACACCC
vGlut1 R	CATGAGCTTGGCGCTTTCTC
vGat F	ACACCGGCAAGATCCTCATC
vGat R	GCCACATACGAGTCCCTCAC

### Preparation of snRNA-seq Samples

The DNBelab C Series Single-Cell Library Prep Set (MGI, 1000021082) was used as previously described [27]. Briefly, single**-**nucleus suspensions were prepared for droplet generation, emulsion breakage, bead collection, reverse transcription, and cDNA amplification to create barcoded libraries. Indexed libraries were then constructed according to the manufacturer’s protocol. The sequencing libraries were quantified using the Qubit ssDNA Assay Kit (Thermo Fisher Scientific). Sequencing was performed on the DIPSEQ-T7 sequencer at BGI-Beijing, employing a 41-bp read length for read 1 and a 100-bp read length for read 2.

### Sequencing Data Processing

Raw sequencing reads generated by DIPSEQ-T7 were filtered and demultiplexed using PISA (v.0.7) (https://github.com/shiquan/PISA). The filtered reads were then aligned to the reference genome mRatBN7.2 using STAR (v2.7.3a) [28]. For tissues sequenced with snRNA-seq, transcripts of genes included both exon and intron reads [29], as unspliced pre-mRNAs were abundantly present in the cell nucleus. Finally, PISA was used to generate a nucleus-gene UMI count matrix.

### Clustering and Annotation of Cells

DoubletFinder [30] was used to remove doublets from each library via default parameters, excluding 5% of cells resembling pseudo-doublets. Nuclei with fewer than 500 detected genes, 1,000 unique molecular identifiers, or 10,000 reads were filtered out. Additionally, cells or nuclei with mitochondrial gene counts exceeding 10% were excluded. Unsupervised clustering was performed using Seurat (4.3.0.1) [31] in R (v4.3.1). Libraries from the same tissue were merged, and the ‘NormalizeData’ and ‘FindVariableGene’ functions were applied to identify the top 3,000 most variable genes using the “vst” method. The merged data from each brain region were scaled, followed by principal component analysis (PCA). Batch effects were corrected using Harmony (v0.1.1) [32], and Uniform Manifold Approximation and Projection (UMAP) was employed for dimension reduction based on the top 20 principal components. Finally, the FindClusters function was used to identify cell clusters at a resolution of 0.5. Cell types were defined using classical markers and cell markers from published studies.

### Differential Abundance (DA) Testing

We used miloR (v1.2.0) [33] to perform DA testing, individually comparing the acute, latent, and chronic phases of each cell type with the control group. For KNN refinement, we used 20 reduced dimensions from Harmony with k = 20 and randomly sampled 5% of the cells to start. Neighborhoods with homogeneity below 0.7 were classified as mixed. A Spatial FDR < 0.1 was considered indicative of a significant difference.

### Differentially Expressed Genes (DEGs) and Gene Enrichment Analysis

Libra (v1.0.0) [34] was used to perform DEG analysis with parameters “de_family = 'pseudobulk', de_method = 'DESeq2', de_type = 'LRT'”. The criteria for identifying upregulated genes were adjusted *P* values < 0.05 and log_2_fold changes > 0.25, while downregulated genes were defined by adjusted *P* values < 0.05 and log_2_fold changes < −0.25. Gene function enrichment was performed using Metascape [35]. Time-trend clustering of DEGs was conducted with Mfuzz (2.64.0) [36], employing default parameters.

### Cell-Cell Communication Analysis

CellChat (v2.1.2) [37] was used to infer and quantify cell-cell communication in this study. The ligand-receptor interaction mouse database from CellChatDB (within CellChat) served as the reference for the cell-cell interaction analysis. The functions ‘identifyOverExpressedGenes’ and ‘identifyOverExpressedInteractions’ were applied with default parameters to identify overexpressed ligand-receptor interactions. The communication probability and cellular communication network were inferred via the ‘CommunProb’ and ‘filterCommunication’ functions with parameters of ‘population’. Size = TRUE, raw. Use = TRUE, and min. Cells = 10’. Then, NicheNet (v2.1.0) was used to infer ligand-to-target signaling pathways based on the mouse dataset [38].

### Trajectory Inference of Astrocytes

CytoTRACE (v.0.3.3) [39] was employed to predict developmental potential using default parameters. Monocle 3 (v1.3.5) [40] was used to conduct trajectory inference analysis in this study. ‘GetAssayData’ and ‘preprocess_cds’ were applied for data preprocessing, and ‘align_cds’ was used to eliminate batch effects from the libraries. For unsupervised clustering based on PCA, the ‘reduce_dimension’ and ‘cluster_cells’ functions were used with default parameters. The trajectory graph was constructed using the ‘learn_graph’ function with the parameter ‘close_loop = F’. Finally, the ‘order_cells’ function was used to arrange cells according to their developmental potential, as predicted by CytoTRACE. ‘Graph_test’ was used to identify genes related to differentiation, with a *P*-value < 0.01 and Moran’s I > 0.25 as the criteria. Additionally, ClusterGVis (v0.1.1) was used to cluster and visualize differentiation-related genes using the elbow method.

### Experimental Design and Statistical Analysis

Rats were randomly divided into four groups: control (with saline injection), 24 h (Acute), 7 d (Latent), and 8 w (Chronic) following SE. Each group was designed to consist of 9-11 rats, except for the control and chronic groups, which included 12-14 rats, as three of them were intended for EEG recordings. The rats subjected to EEG recordings in the fourth week post-SE were excluded from subsequent experiments, as electrode implantation was anticipated to influence gene expression. Four to eight rats per group were used for qRT-PCR, and three individual rats from each group served as biological replicates for snRNA-seq analysis.

All the data are presented as the means ± SD. Statistical analyses were performed with GraphPad Prism 10.1.2 (GraphPad Software). For qRT-PCR analysis, all data passed the normality test (Shapiro–Wilk test) and variance equality. Comparisons among multiple groups of parametric data were performed using one-way ANOVA. A *P*-value <0.05 was considered to indicate statistical significance.

## Results

### Pilocarpine-Induced Rat Model of Temporal Lobe Epilepsy

To comprehensively investigate the cellular and molecular changes occurring during epileptogenesis, we first established a PILO-induced epilepsy model in rats. Following the intraperitoneal injection of PILO, the rat model exhibited the typical behavioral characteristics of the five seizure stages on the Racine scale [24]: Stage 1, mouth and facial movements; Stage 2, head nodding; Stage 3, forelimb clonus; Stage 4, rearing; and Stage 5, rearing and falling. We used video behavioral monitoring, electroencephalography (EEG), histological staining, and qRT-PCR to confirm the successful establishment of the epilepsy model.

**Fig. 1:**
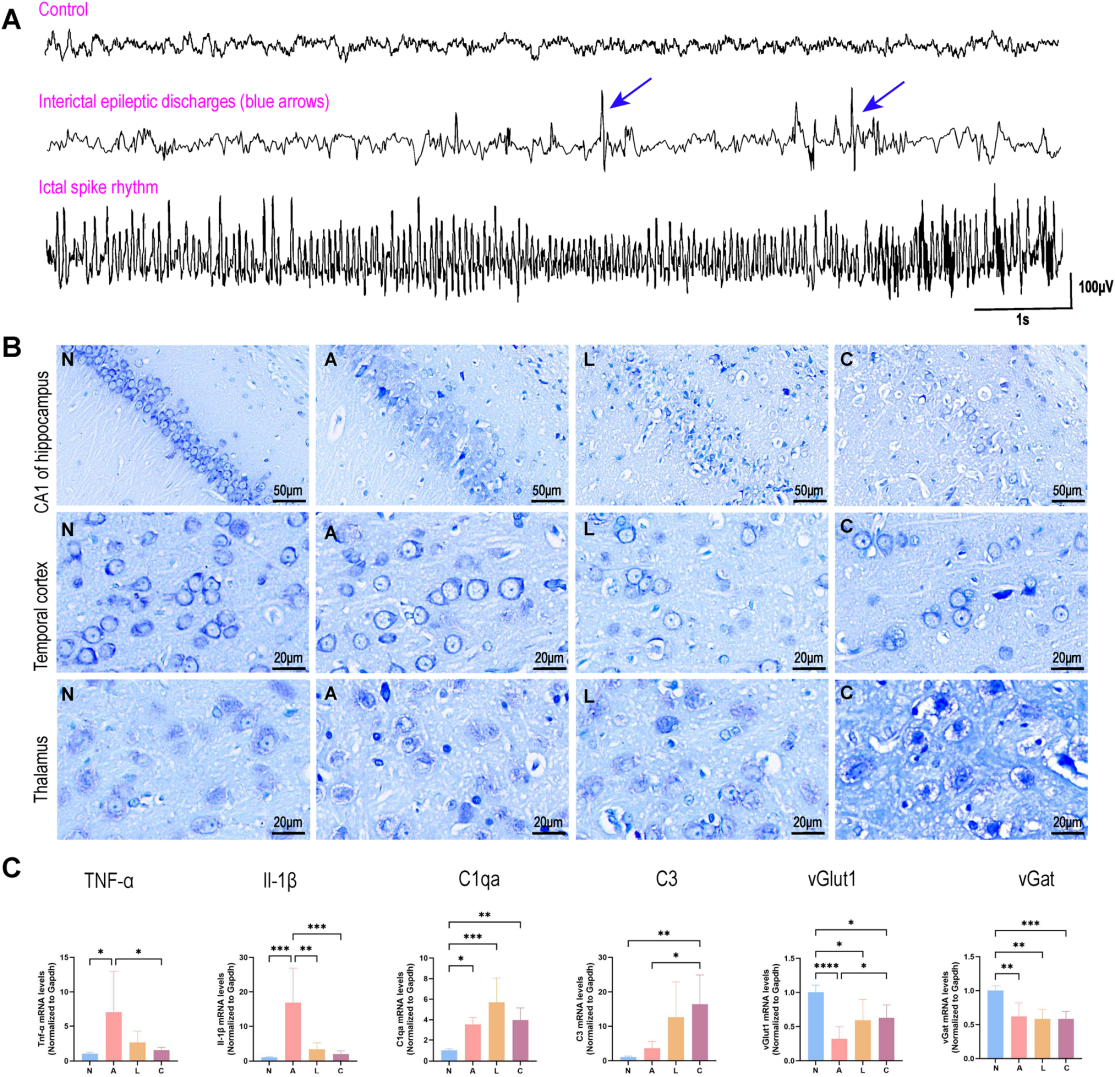
Establishment of the pilocarpine-induced epilepsy rat model. **A** EEG recordings taken in the fourth week after pilocarpine injection show interictal epileptic discharges and spontaneous seizures. The blue arrows in the second episode highlight spike waves, in contrast to the control group (first episode). Epileptic rats exhibited spontaneous seizures characterized by rhythmic spike waves with a frequency >2 Hz and an amplitude three times the baseline (third episode). **B** Nissl staining was performed on the CA1 region of the hippocampus, the temporal cortex, and the thalamus in the control group and the three phases. Neuronal loss was persistent in the CA1 region throughout all phases and was particularly pronounced during the acute phase. The temporal cortex and thalamus also exhibited slight neuronal death, with notable atrophy observed in the thalamus, especially in the chronic phase. **C** The relative mRNA expression levels of *Tnf-α* (*F* value=4.221), *Il-1β* (*F* value=11.43), *C3* (*F* value=7.043), *C1qa* (*F* value=11.36), *vGlut1* (*F* value=12.61), and *vGa*t (*F* value=9.853) in the hippocampus were compared across three phases with those in the control group (*n*=4–8 per group). The *P*-value was calculated using one-way ANOVA. * *P <* 0.05, ** *P <* 0.01, *** *P <* 0.001, **** *P <* 0.0001. **N,** control group; **A**, acute phase; **L,** latent phase; **C,** chronic phase. Astro-astrocytes, Micro-microglia, EX-excitatory neuron, IN-inhibitory neuron, OL-oligodendrocyte, OPC-oligodendrocyte precursor cell, Endo-endothelial cell.

Specifically, only seven rats were included in the chronic group after eight weeks of video behavioral monitoring (Table S1). The average latency period was 21.43 ± 7.39 d, with an average frequency of SRSs of 0.59 ± 0.93 times per day and an average SRS duration of 38.57 ± 10.42 s. None of the rats in the control group developed SE or SRSs. EEG recordings performed in the fourth week post-SE revealed interictal epileptic discharges and ictal spike rhythms during SRSs in epileptic rats, but not in control rats, using the same batch of animals employed for snRNA-seq (Fig. 1A). Histological analysis via Nissl staining demonstrated persistent neuronal death in the CA1 region of the hippocampus during epileptogenesis, with more pronounced neuronal loss in the acute phase. Additionally, a slight decrease in temporal cortex neurons and atrophy in the thalamus were observed, particularly during the chronic phase (Fig. 1B). These findings align with previous observations in TLE epilepsy models [15].

At the molecular level, significant inflammatory activation was observed, with mRNA expression levels of inflammatory factors *Tnf-α* and *Il-1β* significantly increased in the acute phase (Fig. 1C). Complement activation was also noted, with mRNA levels of complement *C3* and *C1qa* upregulated during epileptogenesis, particularly in the chronic phase (Fig. 1C). Furthermore, synaptic loss was observed, with decreased mRNA levels of the excitatory synapse marker *vGlut1* and the inhibitory synapse marker *vGat* in the epilepsy group during epileptogenesis (Fig. 1C). These findings are consistent with previous studies [8, 41, 42]. Thus, we confirm that we have successfully established a rat TLE model via intraperitoneal PILO injection.

### Single-Nucleus Transcriptomic Atlas of a Pilocarpine Rat Model of Epileptogenesis

**Fig. 2:**
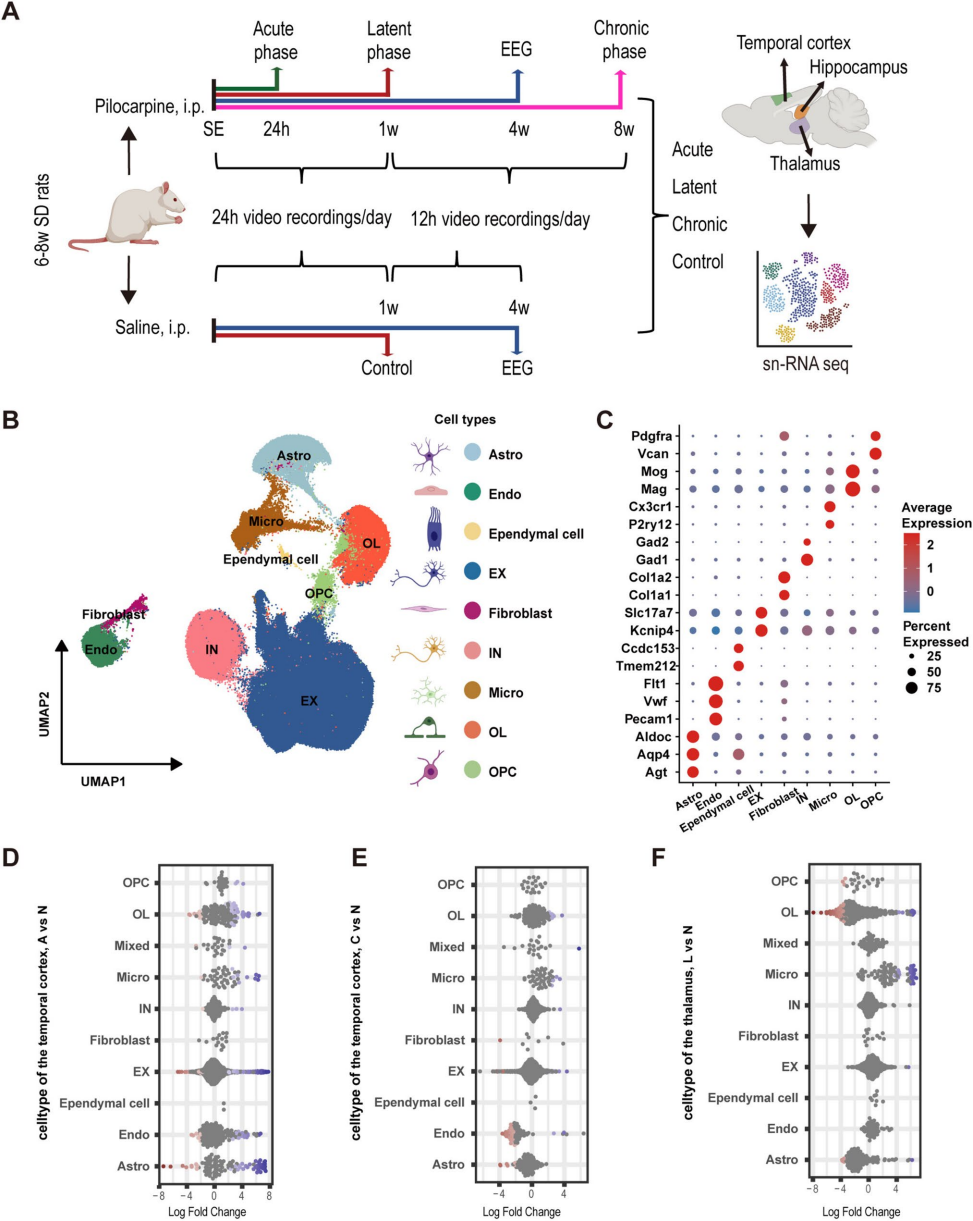
Single-nucleus transcriptomic atlas of the pilocarpine rat model during epileptogenesis. **A** The hippocampus, temporal cortex, and thalamus of rats from both the control group and the three phases were collected at defined time points. Three individual rats were used as biological replicates for each group, resulting in a total of 36 samples for snRNA-Seq analysis. Continuous 24-hour video recordings were conducted on all animals during the first seven days post-SE to ensure that none of the rats exhibited spontaneous seizures during the latent phase. Beginning on the 8th day after modeling, surviving animals were monitored via continuous video for 12 h daily over the next 7 weeks to detect SRSs. EEG recordings were conducted on a subset of animals from the same experimental batch used for snRNA-Seq in the fourth week and were excluded from subsequent experiments. **B** UMAP representation of 311,177 nuclei from 36 samples, showing nine clusters, each represented by a different color. **C** Dot plots displaying the marker genes for each cluster. **D**–**F** Changes in cell abundance in the temporal cortex during the acute (**D**) and chronic (**E**) phases, as well as in the thalamus during the latent phase (**F**), compared to the control group. Neighborhoods that overlap with the same cell population are grouped together and colored to indicate significant differential abundance (DA) (Spatial FDR < 0.1). Cell clusters with significantly decreased abundance are shown in red, while those with significantly increased abundance are shown in blue. **N**, control group; **A,** acute phase; **L**, latent phase; **C**, chronic phase. Astro-astrocytes, Micro-microglia, EX-excitatory neuron, IN-inhibitory neuron, OL-oligodendrocyte, OPC-oligodendrocyte precursor cell, Endo-endothelial cell.

With the established rat epilepsy model, we conducted a single-nucleus transcriptomic analysis. Single-nucleus sampling focused on four groups of rats: the control group (7 d after receiving an equal volume of saline as a substitute for PILO), the acute phase group (24 h post-SE), the latent phase group (7 d post-SE), and the chronic phase group (8 w post-SE) of epileptogenesis (Fig. 2A). Three individual rats were used as biological replicates for each group. For the twelve animals used for snRNA-Seq, the average dose of pilocarpine administered in the epilepsy group was 23.33±5.00 mg/kg, while the average saline dose in the control group was 23.33 ± 5.78 mg/kg. The total doses for the experimental and control rats averaged 34.33 ± 5.00 mg/kg and 34.33 ± 5.77 mg/kg, respectively. The latency to SE (time from initial injection to SE onset) averaged 31.78±4.13 min in the epilepsy group, consistent with previous studies [11] (Table S2).

Tissue samples were collected from three brain regions (the hippocampus, temporal cortex, and thalamus) of each rat, resulting in a total of 36 samples. SnRNA-seq was performed on these samples, and after quality control, we obtained a total of 311,177 single nuclei. These nuclei were annotated into nine cell types (Fig. 2B, C), including astrocytes, microglia, excitatory neurons (EXs), inhibitory neurons (INs), oligodendrocytes (OLs), ependymal cells, oligodendrocyte precursor cells (OPCs), fibroblasts, and endothelial cells. Detailed information on the rats used for snRNA-seq and cell types for each sample is provided in Table S2.

We further analyzed cell type abundance changes during epileptogenesis and observed notable alterations (Fig. 2D–F and Table S3). In the acute phase, the temporal cortex exhibited significant shifts in the abundance of various cell types, reflecting dynamic cellular activity (Fig. 2D). During the chronic phase, we identified a reduction in endothelial cells and astrocytes, accompanied by an increase in OLs and microglia in the temporal cortex (Fig. 2E). In the latent phase, the thalamus showed an increased abundance of microglia, a decreased abundance of OPCs and astrocytes, along with alterations in OLs (Fig. 2F). These findings underscore dynamic cellular responses across different brain regions during epileptogenesis, with the most prominent changes occurring in the temporal cortex during the acute phase.

### Differentially Expressed Genes (DEGs) During Epileptogenesis

**Fig. 3:**
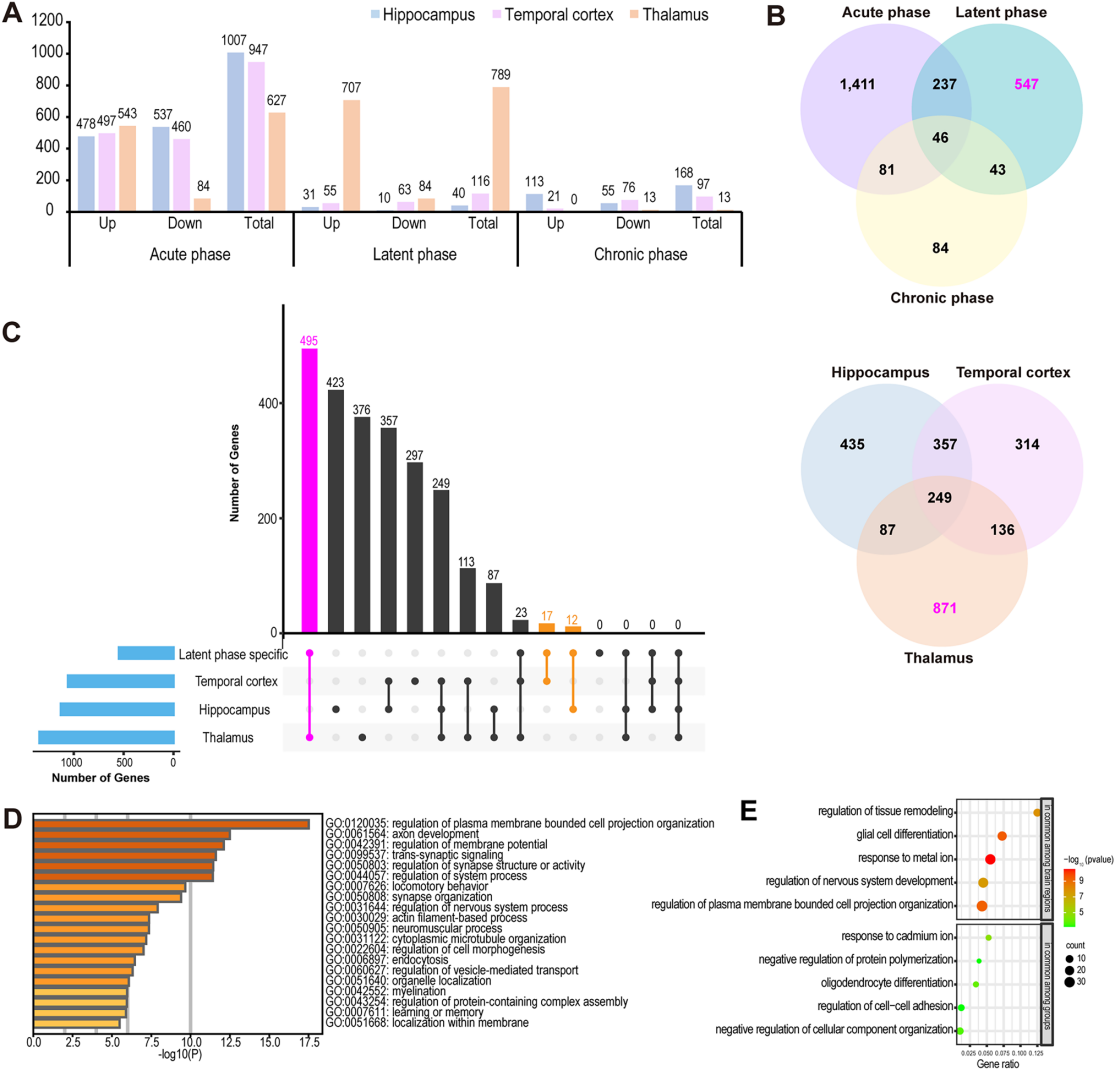
Differentially expressed genes (DEGs) during epileptogenesis. **A** Bar plot showing the number of upregulated, downregulated, and total DEGs in the hippocampus, temporal cortex, and thalamus across the three phases. Upregulated genes were defined as those with adjusted *P* values < 0.05 and log_2_fold changes > 0.25, while downregulated genes were defined as those with adjusted *P* values < 0.05 and log_2_fold changes < −0.25. **B** Venn diagrams (http://www.ehbio.com/test/venn) illustrating the similarities and differences among DEGs identified across the three phases and brain regions. A total of 46 genes were differentially expressed in all three phases, while 249 genes were differentially expressed across all three regions. **C** Region-specific genes and latent phase-specific (547) genes were further analyzed. The thalamus exhibited the greatest number of common genes (495) with latent phase-specific genes, compared to the hippocampus (12) and temporal cortex (17). **D** Functional enrichment analysis of the 495 common genes shared between the thalamus and latent phase-specific genes. **E** Functional enrichment analysis of the 249 genes that were differentially expressed across all three brain regions as well as the 46 genes that were differentially expressed in all three phases.

Building on our investigation of dynamic cellular changes, we further explored the molecular and genetic alterations that occur during epileptogenesis. We compared the gene expression levels in each cell type of the epilepsy groups (across three brain regions and three phases) to those of the corresponding cell type in the control group, identifying a total of 2,449 DEGs in different brain regions during epileptogenesis (Fig. 3A–C, Table S4, and Fig. S1). The acute phase exhibited the highest number of DEGs across all brain regions (1,775), with the hippocampus showing the greatest number (1,007, compared with 947 in the temporal cortex and 627 in the thalamus) (Fig. 3A). This highlights that molecular changes in the hippocampus are most prominent during the onset of epileptogenesis. As epileptogenesis progressed to the latent phase, the thalamus exhibited the highest number of DEGs (789, compared with 40 in the hippocampus and 116 in the temporal cortex) (Fig. 3A, B). Notably, 707 of the 789 DEGs in the thalamus were upregulated. Finally, in the chronic phase, the number of DEGs in the thalamus decreased substantially compared to the latent phase (13 compared with 789), while the number of DEGs in the hippocampus increased compared to the latent phase, though still lower than in the acute phase (168 compared with 40 and 1,007, respectively) (Fig. 3A, B). These findings suggest that, similar to the acute phase, the hippocampus may also serve as the primary site of molecular changes during the chronic phase of epileptogenesis.

Furthermore, we compared the similarities and differences between DEGs identified across the three phases and brain regions (Fig. 3B). The acute phase had the greatest number of unique DEGs (1,411), whereas 46 genes were differentially expressed in all three phases. These genes could serve as molecular markers for identifying epileptogenesis. Genes with phase-specific differential expression could also act as molecular signatures distinguishing different disease stages. Additionally, we identified 249 genes that were differentially expressed across all three brain regions, with 435, 314, and 871 genes exclusively expressed in the hippocampus, temporal cortex, and thalamus, respectively (Fig. 3B, C). Region-specific analysis revealed that among 547 genes specific to the latent phase, the thalamus had the largest subset (495), compared with the hippocampus (12) and temporal cortex (17) (Fig. 3C), further implicating the thalamus as a major site of molecular changes during the latent phase. Functional enrichment analysis of these 495 genes (Fig. 3D) identified enriched pathways, such as regulation of membrane potential, trans-synaptic signaling, and synapse organization. Taken together, these findings suggest that the thalamus may play a significant role in synaptic remodeling during the latent phase.

In addition, to elucidate the roles of the 46 genes differentially expressed across all phases and the 249 genes expressed across all regions, we performed functional enrichment analysis on these genes (Fig. 3E). The 249 genes expressed across all brain regions were enriched in pathways related to glial differentiation, neuronal remodeling, and synaptic remodeling. Several of these genes, including *Gfap* [43], *Stat3*, *Atp1b2*, *Eef2k* [44], *S100b* [45], *Spp1*, *Agt*, *Cd14*, and *Igfbp* [46], have been implicated in human epilepsy or animal models. Meanwhile, the 46 genes altered across all three phases were associated with metabolism, biosynthesis, and cell-cell adhesion. Some of these genes, such as *Cnn3* [47], *Cst3* [48], *Gjb6* [49], *Hspb1* [50], and *Kcnip2* [51], have been previously reported in epilepsy studies.

In summary, these findings suggest that glial differentiation, neuronal remodeling, and synaptic remodeling processes occur in the hippocampus, temporal cortex, and thalamus during epileptogenesis, while metabolic and biosynthetic processes play roles at different stages of the disease.

### Critical Genes Involved in Epileptogenesis

**Fig. 4:**
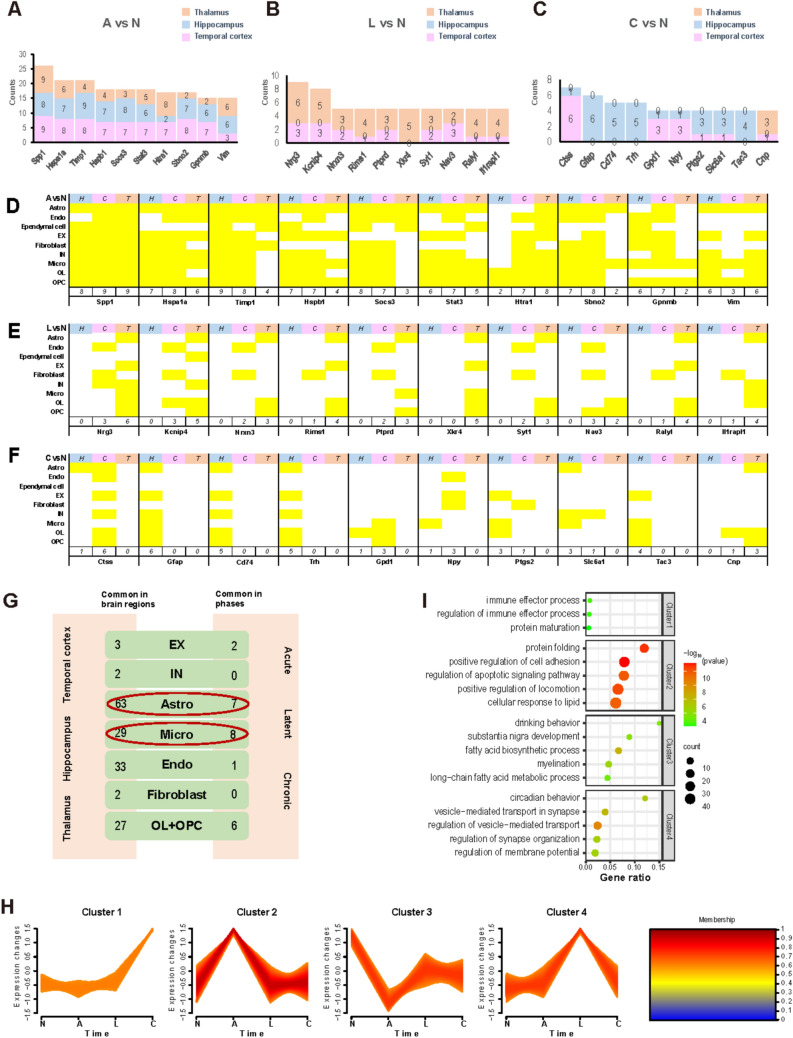
Key differentially expressed genes (DEGs) and four major gene clusters involved in epileptogenesis. The top 10 DEGs in nine cell types across the acute (A), latent (L), and chronic phases (C) compared with those in the control group (N) are presented in bar plots **A**, **B**, and **C**. The number of cell types is labeled on the bar plots, with detailed cell types illustrated in **D, E,** and **F,** respectively. **G** The number of common genes identified in each cell type across the three brain regions and three phases. **H** Time-trend clustering of DEGs. Four cluster categories were identified based on their gene expression patterns among the control, acute, latent, and chronic phases: Cluster 1, genes significantly upregulated in the chronic phase. Cluster 2: Genes significantly upregulated in the acute phase. Cluster 3: Genes significantly downregulated in the acute phase. Cluster 4: Genes significantly upregulated in the latent phase. **I** Gene functional enrichment analysis of the four clusters highlights their respective biological roles during different phases of epileptogenesis.

To reveal comprehensive and specific molecular changes in the development of epileptogenesis, we further identified critical genes that may be involved in this process. By comparing the three phases with the control, we calculated the number of cell types in which the identified DEGs showed differential expression and listed genes that exhibited differential expression across most cell types. In the acute phase, genes including *Spp1*, *Hspa1a*, *Timp1*, *Hspb1*, *Socs3*,* Stat3*, *Htra1*, *Sbno2*, *Gpnmb*, and *Vim* were differentially expressed across most cell types (Fig. 4A), with specific cell types detailed in Fig. 4D. Among these key genes, those related to inflammation, such as *Spp1*, *Socs3*, *Stat3*, *Hsp*s, and *Sbno2*, may play roles in sensing stress and transmitting stress signals to downstream genes, while *Htra1, Gpnmb, Timp1*, and *Vim* reflect dramatic structural changes in various cell types, embodying the two key features of molecular alterations in the acute phase. Compared with the acute phase, fewer genes were differentially expressed in multiple cell types during the latent and chronic phases. In the latent phase, *Nrg3, Kcnip4, Nrxn3, Rims1, Ptprd, Xkr4, Syt1, Nav3, Ralyl,* and *Il1rapl1* were the most frequently identified DEGs across cell types (Fig. 4B, E), primarily expressed in the thalamus. These genes are involved in electrical signal generation and conduction (*Kcnip4, Syt1,* and *Nav3*), as well as cell migration and proliferation (*Nrg3, Ptprd, Nrxn3, Ralyl,* and *Rims1*), which may relate to synaptic remodeling. Finally, in the chronic phase, the key genes included *Ctss, Gfap, Cd74, Trh, Gpd1, Npy, Ptgs2, Slc6a1, Tac3,* and *Cnp* (Fig. 4C, F), mainly expressed in the hippocampus. These genes are associated with immune regulation (*Ctss, Cd74, Gfap,* and *Ptgs2*), neural regulation (*Npy, Slc6a1,* and *Cnp*), and metabolism regulation (*Gpd1, Trh,* and *Tac3*). In conclusion, during the acute and chronic phases, gene expression differences were most evident across cell types in the hippocampus, whereas differences in some genes were less pronounced in cells of the temporal cortex and thalamus. However, during the latent phase, expression differences were primarily observed in thalamic cell types, suggesting significant genetic alterations in the thalamus during this period.

We further analyzed changes in DEGs across various cell types. Specifically, for each cell type (OLs and OPCs grouped together) in different brain regions, we compared them to the control group across the three phases of epileptogenesis to identify DEGs. Our focus was on genes that consistently showed differential expression across all three phases (Fig. S2 and Table S5) within each cell type, as well as those shared across different brain regions (Fig. S3 and Table S5), as these genes are more likely to be strongly related to epileptogenesis. The total number of such common genes is summarized in Fig. 4G, with specific genes detailed in Table S5. Gene function enrichment analysis was performed on the common genes across the three brain regions (Fig. S4A) and the three phases (Fig. S4B). Genes commonly altered across brain regions were primarily associated with tissue remodeling, cellular signaling, gliogenesis, immune responses, metabolism, and homeostasis. In contrast, genes commonly altered across phases were associated with cell migration and development, glial cell differentiation, and metabolic responses. Specifically, we identified three genes—*Gjb6*, which was downregulated across the three brain regions and phases of epileptogenesis in astrocytes; *Ankrd55*, which was downregulated; and *Wapl*, which was upregulated across the three brain regions and phases of epileptogenesis in microglia (Fig. 4G, Fig. S5, and Table S5). *Gjb6* encodes Connexin 30 (Cx30), a protein in the connexin family that forms gap junctions between cells. Previous research showed that Cx30 was overexpressed in the hippocampus at both the transcriptional and translational/posttranslational levels at the beginning of kindling epileptogenesis. This overexpression remained at the mRNA level (but not at the protein level) after focal seizures were acquired, but was significantly downregulated in epileptic animals [49]. This suggests that Cx30 plays a crucial role in epileptogenesis. *Ankrd55* is associated with some autoimmune disorders like multiple sclerosis and rheumatoid arthritis [52], while *Wapl* regulates cohesin's release from chromatin by opening a distinct DNA exit gate [53]. Although the roles of *Ankrd55* and *Wapl* in microglia during epileptogenesis are not well understood, our results suggest their potential involvement in this process.

Additionally, we observed that EXs and INs shared few common genes across regions and phases, indicating significant gene expression variability in neurons across different phases and regions during epileptogenesis. In contrast, glial cells, including astrocytes, microglia, and OLs/OPCs, exhibited many shared genes across all three regions, suggesting glial cells may drive similar processes across brain regions. Further analysis of five common genes expressed in glial cells—*Hspa1a, Hspb1, Spp1, Stat3,* and *Vim* (Table S5)—revealed their involvement in inflammation and cytokine signaling pathways, indicating that glial cells may mediate inflammatory responses across all brain regions during epileptogenesis.

### Different Gene Clusters Revealed Functional Changes During Epileptogenesis

To better understand the functions of the 2,449 DEGs identified during epileptogenesis, we classified all DEGs into four gene clusters (Fig. 4H and Table S6) based on their gene expression changes across the control group, acute phase, latent phase, and chronic phase. These clusters included: (1) genes significantly upregulated in the chronic phase (Cluster 1); (2) genes significantly upregulated in the acute phase (Cluster 2); (3) genes significantly downregulated in the acute phase and subsequent phases (Cluster 3); and (4) genes significantly upregulated in the latent phase (Cluster 4). To elucidate the functions of specific genes within each cluster, functional enrichment analysis was conducted (Fig. 4I). Genes significantly downregulated in the acute phase (Cluster 3) were primarily associated with various cellular metabolic processes, as well as cell growth and development. In contrast, genes significantly upregulated in the acute phase (Cluster 2) were related to cell regulation and adhesion, and the apoptotic signaling pathway. These findings indicate that during the acute phase, metabolic synthesis processes, neuronal development, and synaptic maturation are impaired, accompanied by neuronal apoptosis and neuroinflammation. Genes upregulated in the latent phase (Cluster 4) were enriched in pathways associated with synapses and membrane potential, highlighting the latent phase as a critical period for synaptic and neural network remodeling during epileptogenesis. Finally, Cluster 1, which included genes upregulated in the chronic phase, showed enrichment in pathways related to immune processes.

Together, these four distinct clusters of DEGs reveal the major molecular changes occurring across the different phases of epileptogenesis, offering insight into the phase-specific mechanisms driving the disease.

### Cell-Cell Interaction Dynamics Indicate Strong Intercellular Communication During the Latent Phase

**Fig. 5:**
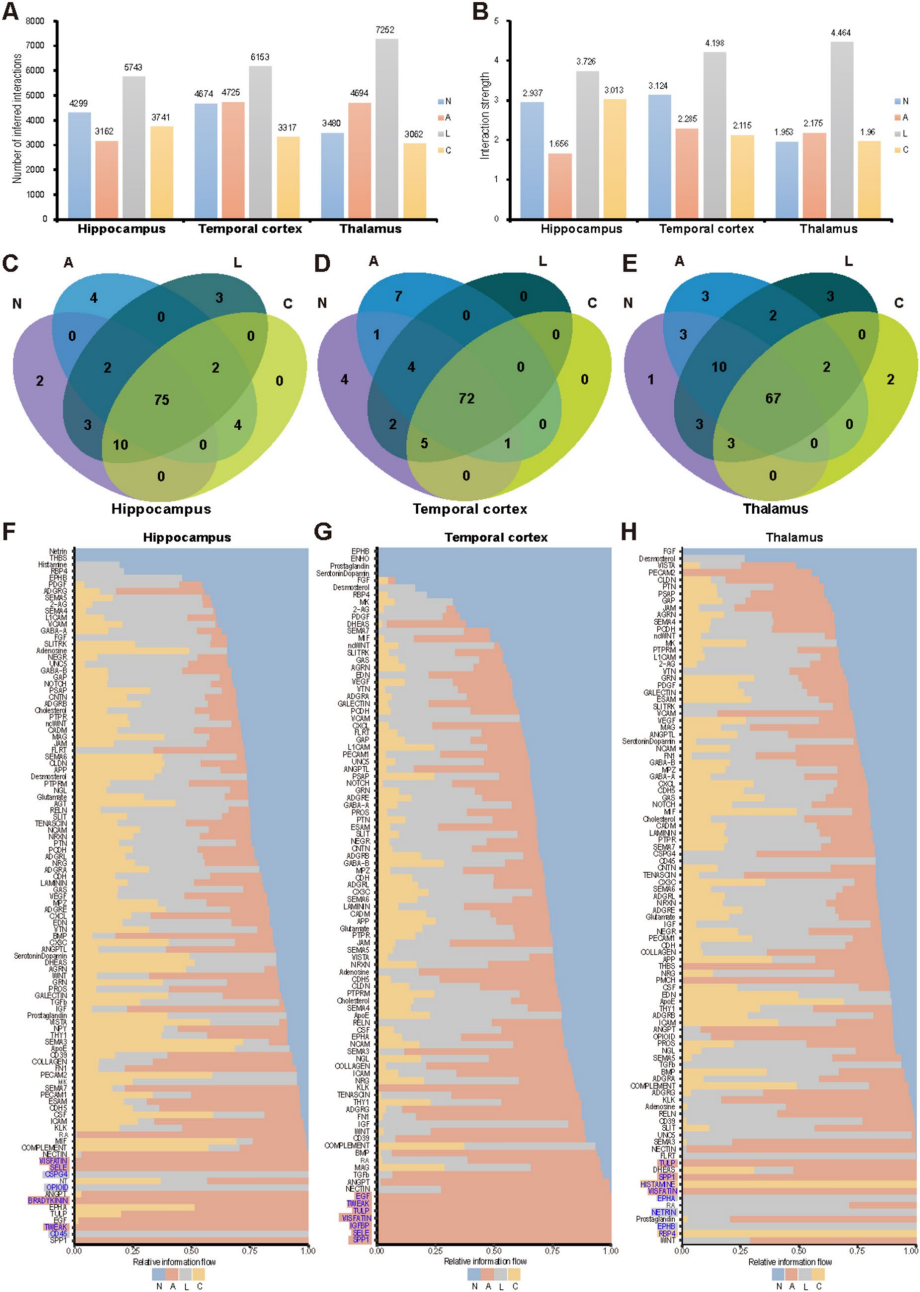
Cell-cell interaction dynamics during epileptogenesis. Number (**A**) and intensity (**B**) of cell interactions across the three brain regions during epileptogenesis. **C**, **D**, and **E** show the number of common and specific cell interaction pathways in the hippocampus (**C**), temporal cortex (**D**), and thalamus (**E**) during different phases. The cell interaction pathways used in the hippocampus, temporal cortex, and thalamus across different phases are detailed in **F**, **G,** and **H**, respectively, with the specific pathways labeled in blue.

Cell-cell interactions play a crucial role in regulating various biological processes [54]. Accordingly, we analyzed the interactions among different cell types during epileptogenesis (Fig. 5 and Table S7). Regardless of the quantity (Fig. 5A) or intensity (Fig. 5B), cell interactions were found to be strongest during the latent phase—a trend that remained consistent across all three brain regions. In contrast, the most pronounced changes in gene expression occurred during the acute phase, particularly in the hippocampus, where cell interactions were at their lowest compared to the control group. However, interactions in the temporal cortex and thalamus showed relative increases compared to the control group during the acute phase.

We then examined the number of cell interaction pathways and their usage across different phases and brain regions (Fig. 5C–H). Specifically, we focused on the unique signaling pathways in the three brain regions at different phases, as highlighted in Fig. 5F–H. To further clarify the specific cell types involved in these unique signaling pathways, we used heatmaps to visualize cell communications among different cell types across the three brain regions during epileptogenesis (Figs. S6–S8). During the acute phase, four exclusive signaling pathways were utilized in the hippocampus: TWEAK, BRADYKININ, SELE, and VISFATIN (Fig. 5C, F). Specifically, the TWEAK and VISFATIN signaling originated from endothelial cells and targeted astrocytes. SELE signaling was directed from endothelial cells to EXs, while BRADYKININ signaling was transmitted from fibroblasts to endothelial cells (Fig. S6). In the temporal cortex, the exclusive pathways included SPP1, SELE, IGFBP, VISFATIN, TULP, TWEAK, and EGF (Fig. 5D, G). Most of these signaling pathways were transmitted from endothelial cells and astrocytes to EXs and astrocytes (Fig. S7). In the thalamus, SPP1, TULP, and VISFATIN were identified as exclusive pathways (Fig. 5E, H). SPP1 originated from EXs and targeted astrocytes and endothelial cells, while TULP and VISFATIN were directed from endothelial cells to OLs and other endothelial cells (Fig. S8). During the latent phase, the hippocampus and thalamus each exhibited three distinct cell interaction signaling pathways. In the hippocampus, these included CD45, OPIOID, and CSPG4 (Fig. 5C, F), with CSPG4 and OPIOID originating from endothelial cells to INs, and CD45 from microglia to fibroblasts (Fig. S6). In the thalamus, the exclusive pathways were EPHB, NETRIN, and EPHA (Fig. 5E, H), all originating from endothelial cells and EXs, and targeting EXs (Fig. S8). In the chronic phase, only the thalamus exhibited two specific pathways: RBP4 and HISTAMINE (Fig. 5E, H). RBP4 originated from fibroblasts and targeted endothelial cells, while HISTAMINE was directed from ependymal cells to EXs (Fig. S8).

Furthermore, we summarized the number of cell communications as well as the dominant senders (sources) and receivers (targets) among different cell types in the three brain regions during epileptogenesis (Fig. S9, 10). Notably, the strongest communications were observed between EXs and INs, with EXs consistently serving as the primary input-output hub for interactive signals. Overall, cell interactions among most cell types were enhanced during the latent phase across the three brain regions. The enhancement of intercellular communication is a key feature of the latent phase and could serve as a potential target for future intervention strategies.

### Two Novel Astrocyte Clusters and the Specific EX-Astro C3-IN Pathway Found in the Acute Phase

**Fig. 6:**
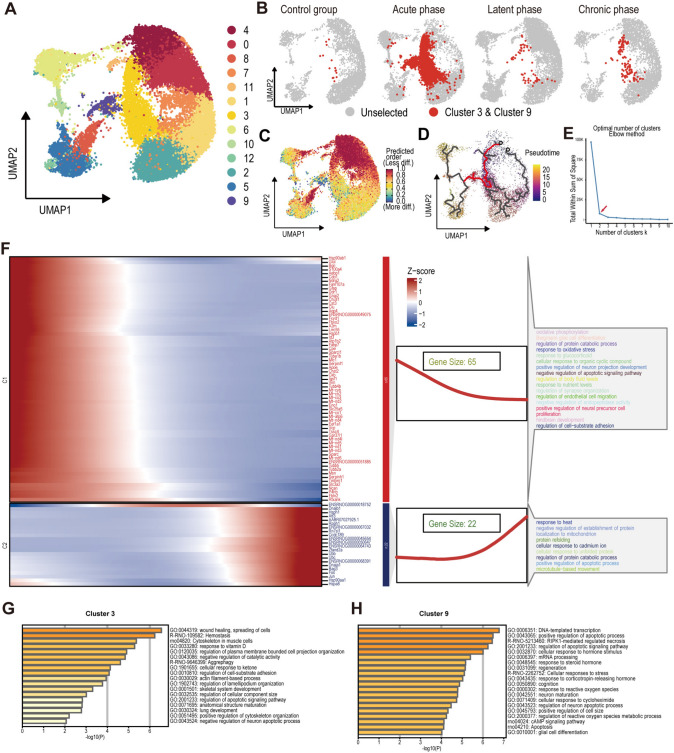
Two novel astrocyte clusters found in the acute phase of epileptogenesis. **A** Subcluster analysis of astrocytes identified a total of 13 subpopulations. **B** Two specific subpopulations, Cluster 3 and Cluster 9, were significantly increased during the acute phase compared to the control group. **C** CytoTRACE was used to predict transformation potential, with red indicating the starting point of transformation and blue indicating the endpoint. Both Clusters 3 and 9 were positioned at the end of the astrocyte trajectory, especially Cluster 9. **D** Trajectories of astrocytes in the acute phase were modeled using Monocle 3, with red lines emphasizing the transformation trajectories toward Cluster 3 and Cluster 9. **E** Cluster analysis of driver genes along the transformation paths to Cluster 3 and Cluster 9 was conducted, and the elbow method determined two optimal subclusters. **F** Heatmaps illustrated dynamic changes in driver genes along the pseudo-time trajectory toward Cluster 3 and Cluster 9. Additionally, functional enrichment analysis results for each clustering group were included. **G**, **H** Gene functional enrichment analysis of genes in Cluster 3 (**G**) and Cluster 9 (**H**).

Accumulating evidence indicates that astrocytes contribute to the development and progression of hyperexcitability in epilepsy due to dysfunctions in gliotransmission, cell metabolism, and immunity [55]. In our single-nucleus analysis of the epileptic brain, we observed notable changes in the number of DEGs in astrocytes during epileptogenesis. Therefore, we further performed subcluster analysis of astrocytes and identified a total of 13 subpopulations (Fig. 6A). Further investigation into astrocytes revealed two distinct clusters (Cluster 3 and Cluster 9) that were significantly increased in the acute phase compared to those in the control group (Fig. 6B and Fig. S11). These clusters remained less abundant in the latent and chronic phases. Notably, they were present in all three brain regions, with the highest abundance in the hippocampus (Fig. S12).

Analysis of their formation process (Fig. 6C) revealed that these clusters are well-developed derivatives of astrocytes, as supported by pseudo-temporal cell trajectory inference (Fig. 6D), which positioned them at the terminal end of the astrocyte trajectory. Potential driver genes associated with their formation were identified through trajectory analysis (Fig. 6E and Table S8). Notable differences in driver genes for the two clusters suggest distinct formation processes (Fig. 6E, F). Cluster 3 showed enrichment for genes related to oxidative phosphorylation, oxidative stress response, cell migration, and synapse organization (Fig. 6F), while Cluster 9 was enriched with genes involved in the heat stress response, calcium ion response, and positive regulation of apoptosis (Fig. 6F). Functional enrichment analysis of genes uniquely expressed in these clusters (Fig. 6G, H) revealed pathways related to glial cell proliferation, indicative of an active proliferative state. Additionally, pathways responding to environmental factors such as external stimuli, hypoxia, polypeptides, and metal ions were enriched, suggesting a role in intercellular signaling and communication. Enrichment in synaptic transmission pathways further pointed to potential roles in regulating neuronal communication.

**Fig. 7:**
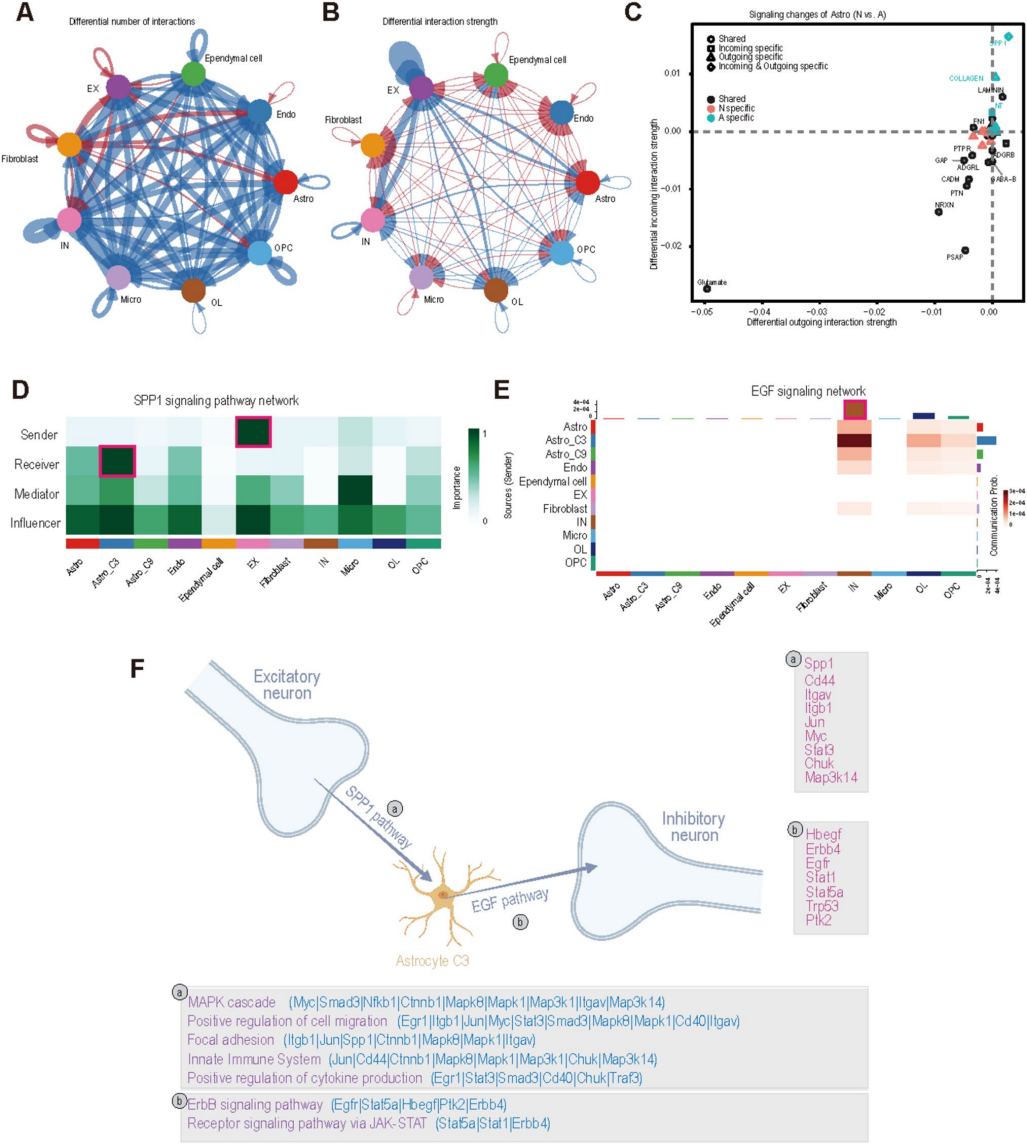
Cell-cell interactions in the hippocampus during the acute phase of epileptogenesis. **A** Differences in the number of cell-cell interactions in hippocampal cells between the acute phase and the control group are shown, with increases highlighted in red and decreases in blue. **B** Differences in the intensity of cell-cell interactions between the acute phase and the control group are visualized, with increases in red and decreases in blue. **C** Differential interaction pathways of astrocytes in the hippocampus between the acute phase and the control group. The SPP1 pathway is identified as a specific input-output pathway. **D** SPP1 pathway network across different cell types. EXs were the primary senders of SPP1 signals, while astrocyte Cluster 3 served as the main receiver. **E** EGF pathway network across different cell types. Astrocytes, particularly Cluster 3, were the primary source of EGF signals, with INs being the main receivers. **F** Schematic diagram of the EX-Astro C3-IN pathway mediated by the SPP1 and EGF pathways in the hippocampus during the acute phase. EXs influence astrocyte C3 via the SPP1 pathway, while C3 further influences INs through the EGF pathway. Critical genes and enrichment functions are listed. The schematic diagram was created using BioRender.com. Astro-astrocytes, Micro-microglia, EX-excitatory neuron, IN-inhibitory neuron, OL-oligodendrocyte, OPC-oligodendrocyte precursor cell, Endo-endothelial cell.

We next examined the cell-cell interactions involving these newly identified clusters to elucidate their functional mechanisms. Focusing on the acute-phase hippocampus, where these clusters were most prominent (Fig. S12), we compared cell-cell interactions between the acute-phase hippocampus group and the control group (Fig. 7A, B, and Table S9). While overall cell interactions decreased in the acute phase, the strength of interactions between astrocytes and several other cell types increased. Notably, several pathways absent in the control group, such as SPP1, COLLAGEN, and NT, emerged in the acute phase. Among these, SPP1-mediated interactions displayed the highest input and output intensities for astrocytes (Fig. 7C). Investigation of the SPP1 pathway across cell types revealed that EXs were the primary senders of SPP1 signals, while astrocyte C3 was the main receiver (Fig. 7D). Interestingly, astrocyte C3 also emerged as the most influenced cells, highlighting a specific influence of EXs on astrocyte C3 via the SPP1 pathway during the acute phase. To explore whether astrocyte C3 influenced other cell types, we analyzed additional acute-phase-specific pathways. The EGF pathway was found to be specifically active, with astrocytes—particularly Cluster 3—serving as the main source of EGF signals, and INs being the primary receivers (Fig. 7E). Based on these findings, we propose that during the acute phase, EXs influence astrocyte C3 through the SPP1 pathway, which subsequently affects INs via the EGF pathway (Fig. 7F). We used NicheNet to infer ligand-to-target signaling pathways based on a mouse dataset. Key genes in the SPP1 pathway included *Spp1, Cd44, Itgav, Itgb1, Cd40, Ctnnb1, Egr1, Jun, Jund, Myc, Nfkb1, Smad3, Stat3, Chuk, Map3k1, Map3k14, Mapk1, Mapk8,* and *Traf3*. Enrichment analysis revealed functions such as the MAPK cascade, positive regulation of cell migration, and focal adhesion (Fig. S13A). Similarly, key genes in the EGF pathway included *Hbegf, Erbb4, Egfr, Stat1, Stat5a, Trp53,* and *Ptk2*, with enrichment functions such as the ErbB signaling pathway and receptor signaling via JAK-STAT (Fig. S13B).

In conclusion, the interaction between EXs, astrocyte C3, and INs represents a prominent change in the hippocampus during the acute phase and may serve as a crucial mechanism underlying acute epileptic seizures. The genes and enrichment functions associated with these pathways could serve as valuable therapeutic targets for future interventions.

## Discussion

This study provides a comprehensive understanding of the dynamic cellular and molecular changes that occur during epileptogenesis, as summarized in Fig. S14. To our knowledge, this study presents the first and most comprehensive single-nucleus transcriptomic dataset covering both temporal aspects (acute phase, latent phase, and chronic phase) and spatial aspects (hippocampus, temporal cortex, and thalamus) of epileptogenesis. First, we concluded that the latent phase is a critical period for synaptic and neural network remodeling during epileptogenesis, suggesting that it represents an opportune time for intervention to potentially prevent epileptic seizures. Furthermore, the thalamus plays a crucial role in this process. Finally, the identification of two novel astrocyte clusters and their role in the acute phase underscores the importance of further research into these clusters and their potential contribution to the pathogenesis of epileptogenesis, particularly the EX-Astro C3-IN pathway in the hippocampus.

We identified a total of 2,449 DEGs, which is consistent with previous studies reporting approximately 2,000 DEGs in TLE [56]. Chen *et al.* [8] used robust rank aggregation analysis to integrate six microarrays from rodent models of TLE. They revealed 146–739 DEGs in the acute phase (119–629 upregulated and 28–117 downregulated), 59–283 DEGs in the latent phase (51–258 upregulated and 1–25 downregulated), and 8–345 DEGs in the chronic phase (4–283 upregulated and 0–62 downregulated) within the rodent hippocampus. However, bulk transcriptomics only provides average gene expression across all cell types [57], whereas our study offers valuable insights into DEGs in distinct cell types. Identifying key genes differentially expressed in various brain regions and across different phases of epileptogenesis offers important clues to potential therapeutic targets. We first examined the top 10 genes differentially expressed in most cell types during the three phases of epileptogenesis (Fig. 4A). In the acute phase, these expression differences were predominantly observed in cell types of the hippocampus and temporal cortex. In contrast, during the latent phase, the differences were primarily observed in cell types within the thalamus, indicating substantial genetic changes in the thalamus. This is particularly significant since few studies have focused on the thalamus during the latent phase, and its role in neural network reorganization during epileptogenesis has been greatly underestimated. Notably, genes related to electrical signal generation and conduction, such as *Kcnip4*, *Syt1*, and *Nav3*, as well as genes involved in cell migration and proliferation, such as *Nrg3*, *Ptprd*, *Nrxn3*, *Ralyl*, and *Rims1*, are promising candidates because of their potential involvement in synaptic remodeling. Targeting these genes may modulate neural network remodeling in the epileptic brain and reveal novel therapeutic targets aimed at halting the progression of epileptogenesis or preventing SRSs. Furthermore, significant gene expression differences in the chronic phase were mainly observed in the cell types of the hippocampus. Interestingly, TLE patients usually exhibit imaging abnormalities in the hippocampus [58], which is clinically recognized as the most common ictogenic zone for TLE seizures [59]. Previous studies suggest that the hippocampus is the most critical site of pathology in epilepsy, likely because most studies have focused on the chronic phase. In our study, however, analysis of DEGs during the latent phase—primarily found in various thalamic cell types, especially glial cells—suggests that the thalamus may play a key role in neural network reorganization during this period. This finding provides new insights into the critical period of epileptogenesis.

Furthermore, we identified genes that exhibit common changes across the three phases and across the three brain regions for each cell type (Fig. S2, S3, and Table S5). We pinpointed three genes that were differentially expressed across the three brain regions and phases of epileptogenesis—*Gjb6, Ankrd55,* and *Wapl*. In addition, we found that EXs and INs exhibited few common genes across different brain regions and phases, whereas glial cells expressed numerous common genes across all three brain regions. These glial genes were mainly related to inflammation and cytokine signaling transduction, suggesting that glial cells drive inflammatory changes across the brain during epileptogenesis. In contrast, neuronal gene expression underwent significant changes across different phases and regions.

In our study, we conducted a detailed analysis of cell-cell interactions across various brain regions during epileptogenesis and delineated specific intercellular signaling pathways present across these regions (Fig. 5F–H and Fig. S14). These pathways could serve as promising intervention targets for future treatment strategies. As noted, the optimal treatment for epilepsy would involve inhibiting the formation of epileptic networks and preventing epileptogenesis [60, 61]; however, preventive treatment remains unavailable [62, 63]. Notably, synaptic pathway enrichment during the latent phase revealed pronounced synaptic and neural network remodeling, accompanied by an overall strengthening of cell interactions. These findings suggest that the latent phase may represent a critical window for intervention to halt the progression of epileptogenesis and potentially prevent spontaneous seizures in the chronic phase, with the thalamus emerging as a key target for therapeutic strategies.

Using single-cell analysis, we identified two novel astrocyte clusters that may represent either subtypes or transient states of astrocytes during epileptogenesis. While future studies are necessary to elucidate their stability, functions, and plasticity, our findings highlight their importance in the progression of epileptogenesis. Functional enrichment analysis indicates that these clusters are actively proliferating and may play critical roles in intercellular signaling, communication, and synaptic transmission. Notably, the functional properties of these clusters align with previous studies on astrocytes in epilepsy [55, 64–66], which demonstrated that astrocytes are activated during the acute phase of epileptogenesis and may influence its progression. Additionally, the interaction between EXs, novel astrocyte C3, and INs via the SPP1 and EGF pathways (Fig. 7F and Fig. S14) may contribute to hyperexcitability during the acute phase and the occurrence of SRSs in advanced epilepsy. Further investigation into the pathological changes related to these astrocyte clusters could determine whether they correspond to the previously described reactive astrocytes and provide deeper insights into the cellular and molecular alterations underlying epileptogenesis, potentially uncovering mechanisms that could inform the development of novel therapeutic targets and strategies.

There are limitations in this study. Most notably, we used a rat model of TLE, which, while providing valuable insights into the cellular and molecular mechanisms underlying epileptogenesis, may not fully capture the complexities of human epilepsy. The rat model was constructed using pilocarpine injection, a widely accepted TLE model [11, 14], which, however, may introduce brain damage that complicates the comparison and identification of key epileptogenesis-related mechanisms. Although previous studies have indicated the involvement of brain damage in epilepsy [11, 14], and numerous studies over the past decades have investigated molecular changes during epileptogenesis using the SE epilepsy model [2, 8, 67–72], distinguishing between features attributable to brain damage and those specific to epileptogenesis remains challenging. Additionally, we did not establish directly comparable control groups for the acute and chronic phases, leaving certain variables, such as differences in age and stress levels, uncontrolled. Despite these limitations, we successfully identified key genes, novel astrocyte clusters, and specific signaling pathways associated with epileptogenesis. These findings open promising avenues for further research and the development of novel therapeutic interventions for epilepsy.

## Supplementary Information

Below is the link to the electronic supplementary material.

Supplementary file1 (XLSX 9 kb)

Supplementary file2 (XLSX 14 kb)

Supplementary file3 (XLSX 2664 kb)

Supplementary file4 (XLSX 903 kb)

Supplementary file5 (XLSX 13 kb)

Supplementary file6 (XLSX 110 kb)

Supplementary file7 (XLSX 3757 kb)

Supplementary file8 (XLSX 593 kb)

Supplementary file9 (XLSX 280 kb)

Supplementary file10 (PDF 1190 kb)

## Data Availability

The data that support the findings of this study have been deposited into the CNGB Sequence Archive (CNSA) [73] of China National GeneBank DataBase (CNGBdb) [74] with accession number CNP0005924.
