# Supplementary material for: Single-Nucleus Transcriptomic Sequencing Revealed Cellular and Molecular Changes in a Pilocarpine-Induced Epilepsy Rat Model
Source: Neurosci Bull. 2025 Jul 24;42(3):539–58. doi: 10.1007/s12264-025-01451-y (PMC12950157; doi:10.1007/s12264-025-01451-y)
Supplement: Supplementary file 10 — Supplementary file10 (PDF 1190 kb) [file 12264_2025_1451_MOESM10_ESM.pdf]

Supplementary Materials Legends

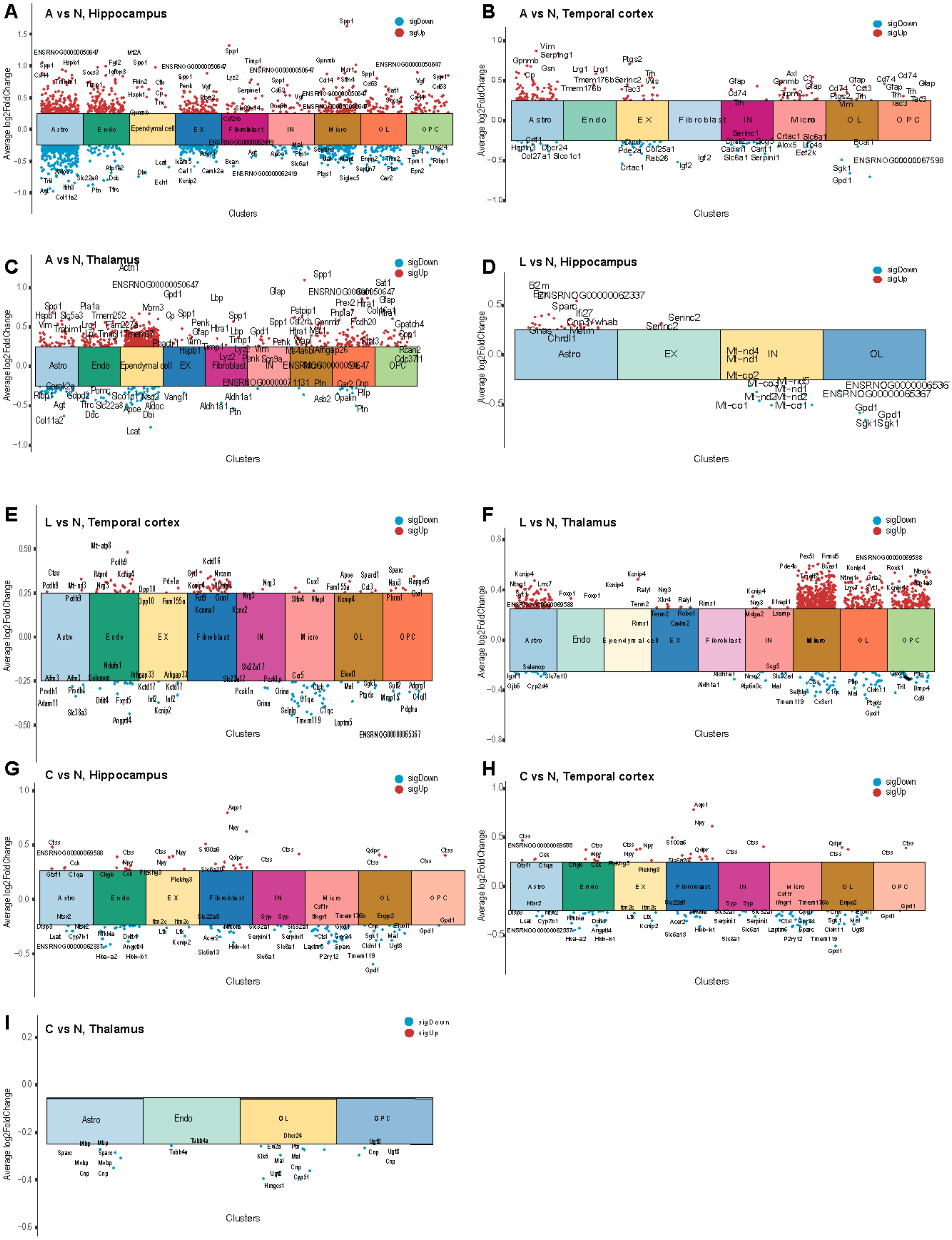

Fig. S1 A-I show differentially expressed genes (DEGs) for each cell type across the acute, latent, and chronic phases in the temporal cortex, hippocampus, and thalamus, compared to the control group. Upregulated genes were defined as those with adjusted *p* values < 0.05 and $\log_2$fold changes > 0.25, while downregulated genes were defined as those with adjusted *p* values < 0.05 and $\log_2$fold changes < -0.25. **N**, control group; **A**, acute phase; **L**, latent phase; **C**, chronic phase. Astro-astrocytes, Micro-microglia, EX-excitatory neuron, IN-inhibitory neuron, OL-oligodendrocyte, OPC-oligodendrocyte precursor cell, Endo-endothelial cell.

A

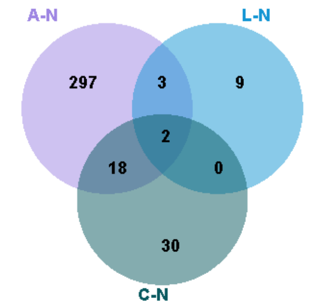

Common genes in EX across the acute, latent, and chronic phases

B

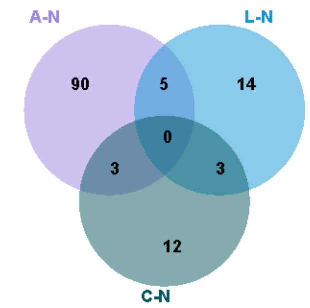

Common genes in IN across the acute, latent, and chronic phases

C

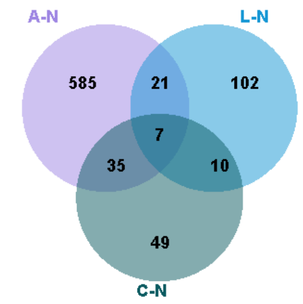

Common genes in astrocyte across the acute, latent, and chronic phases

D

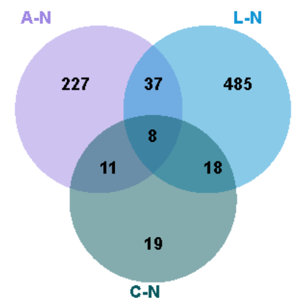

Common genes in microglia across the acute, latent, and chronic phases

E

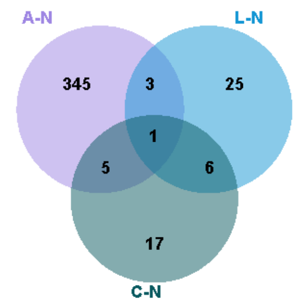

Common genes in Endo across the acute, latent, and chronic phases

F

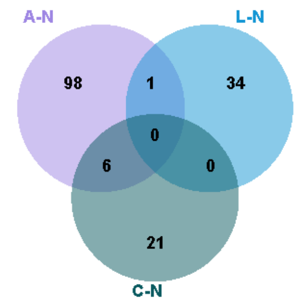

Common genes in fibroblast across the acute, latent, and chronic phases

G

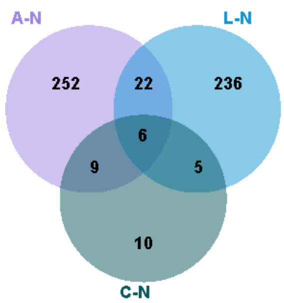

Common genes in OL+OPC across the acute, latent, and chronic phases

Fig. S2 A-G show the number of common differentially expressed genes (DEGs) across the acute, latent, and chronic phases for each cell type. **N**, control group; **A**, acute phase; **L**, latent phase; **C**, chronic phase. Astro-astrocytes, Micro-microglia, EX-excitatory neuron, IN-inhibitory neuron, OL-oligodendrocyte, OPC-oligodendrocyte precursor cell, Endo-endothelial cell.

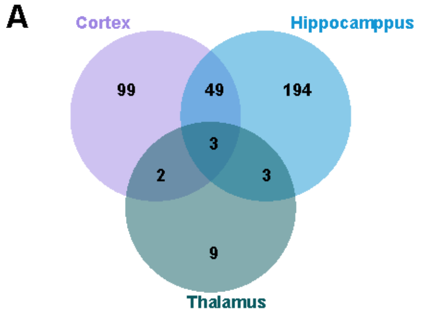

Common genes in EX across the temporal cortex, hippocampus, and the Thalamus.

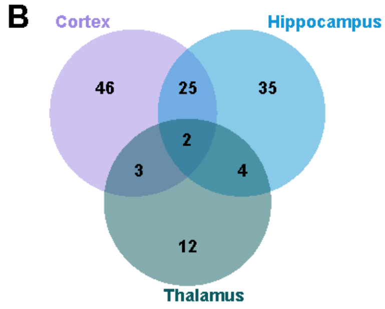

Common genes in IN across the temporal cortex, hippocampus, and the Thalamus.

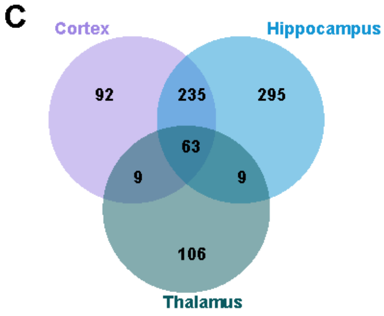

Common genes in astrocytes across the temporal cortex, hippocampus, and the Thalamus.

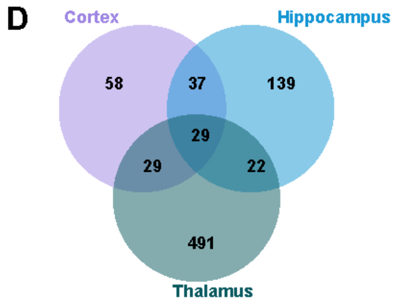

Common genes in microglia across the temporal cortex, hippocampus, and the Thalamus.

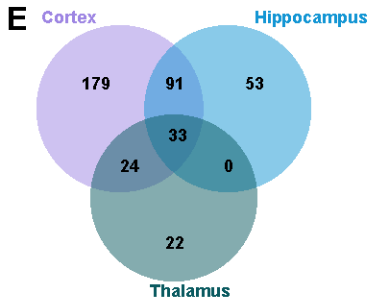

Common genes in Endo across the temporal cortex, hippocampus, and the Thalamus.

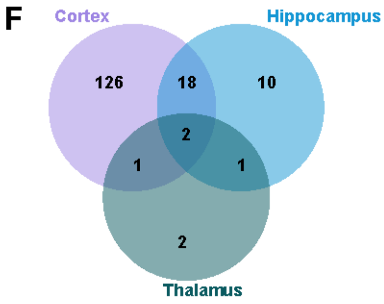

Common genes in Fibroblast across the temporal cortex, hippocampus, and the Thalamus.

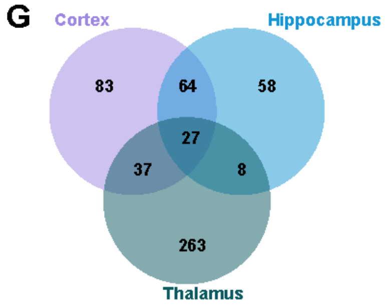

Common genes in OL+OPC across the temporal cortex, hippocampus, and the Thalamus.

Fig. S3 A-G show the number of common differentially expressed genes (DEGs) identified in the temporal cortex, hippocampus, and thalamus for each cell type. **N**, control group; **A**, acute phase; **L**, latent phase; **C**, chronic phase. Astro-astrocytes, Micro-microglia, EX-excitatory neuron, IN-

inhibitory neuron, OL-oligodendrocyte, OPC-oligodendrocyte precursor cell, Endo-endothelial cell.

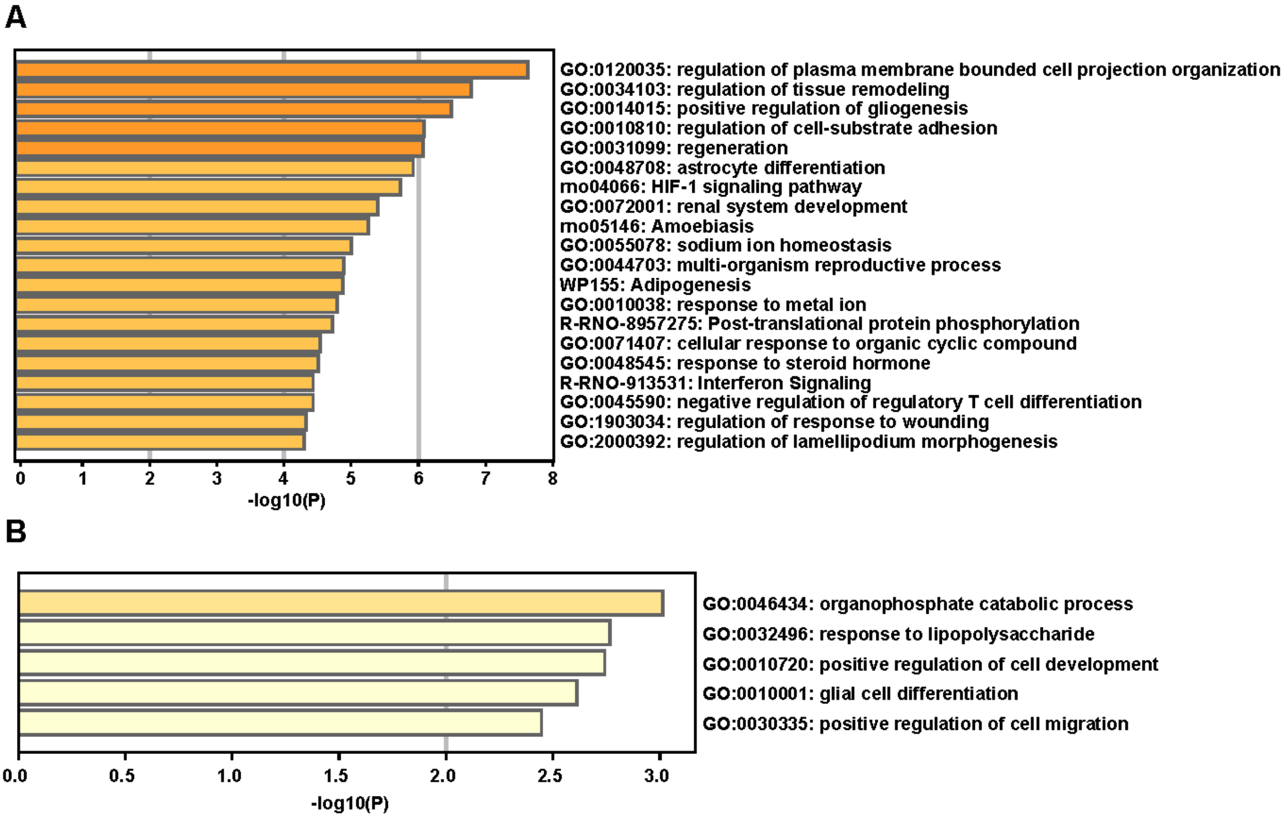

Fig. S4 Gene function enrichment analysis of common genes across the three brain regions (**A**) and the three phases of epileptogenesis (**B**).

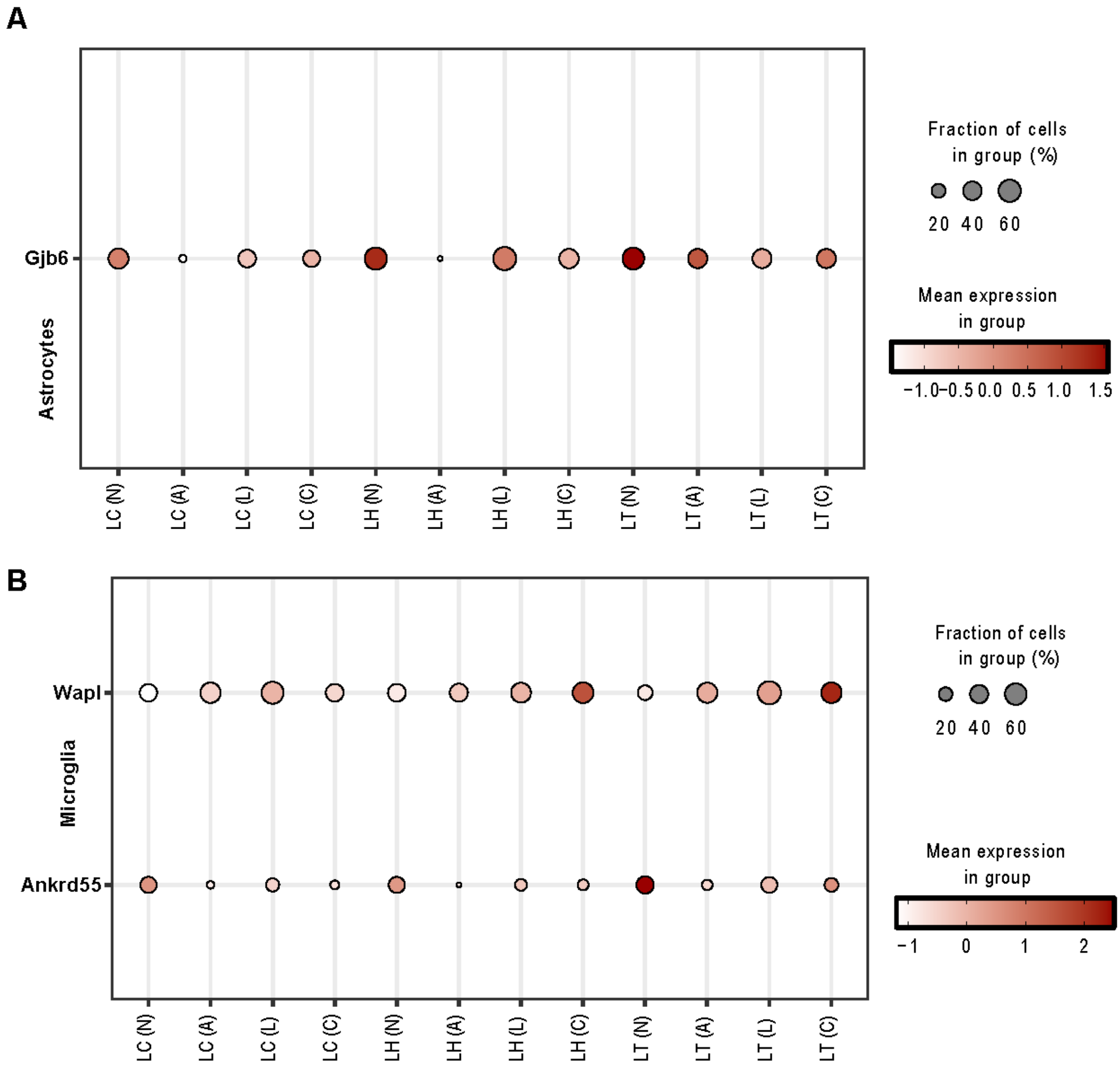

Fig. S5 Three genes were changed across the three brain regions and phases of epileptogenesis in astrocytes (**A**) and microglia (**B**). The color of the dots represents the mean gene expression levels, with higher expression indicated by a darker color. *Gjb6* was downregulated across the three brain regions and phases of epileptogenesis in astrocytes; *Ankrd55*, which was downregulated; and *Wapl*, which was upregulated across the three brain regions and phases of epileptogenesis in microglia. **N**, control group; **A**, acute phase; **L**, latent phase; **C**, chronic phase. LC, temporal cortex; LH, hippocampus; LT, thalamus.

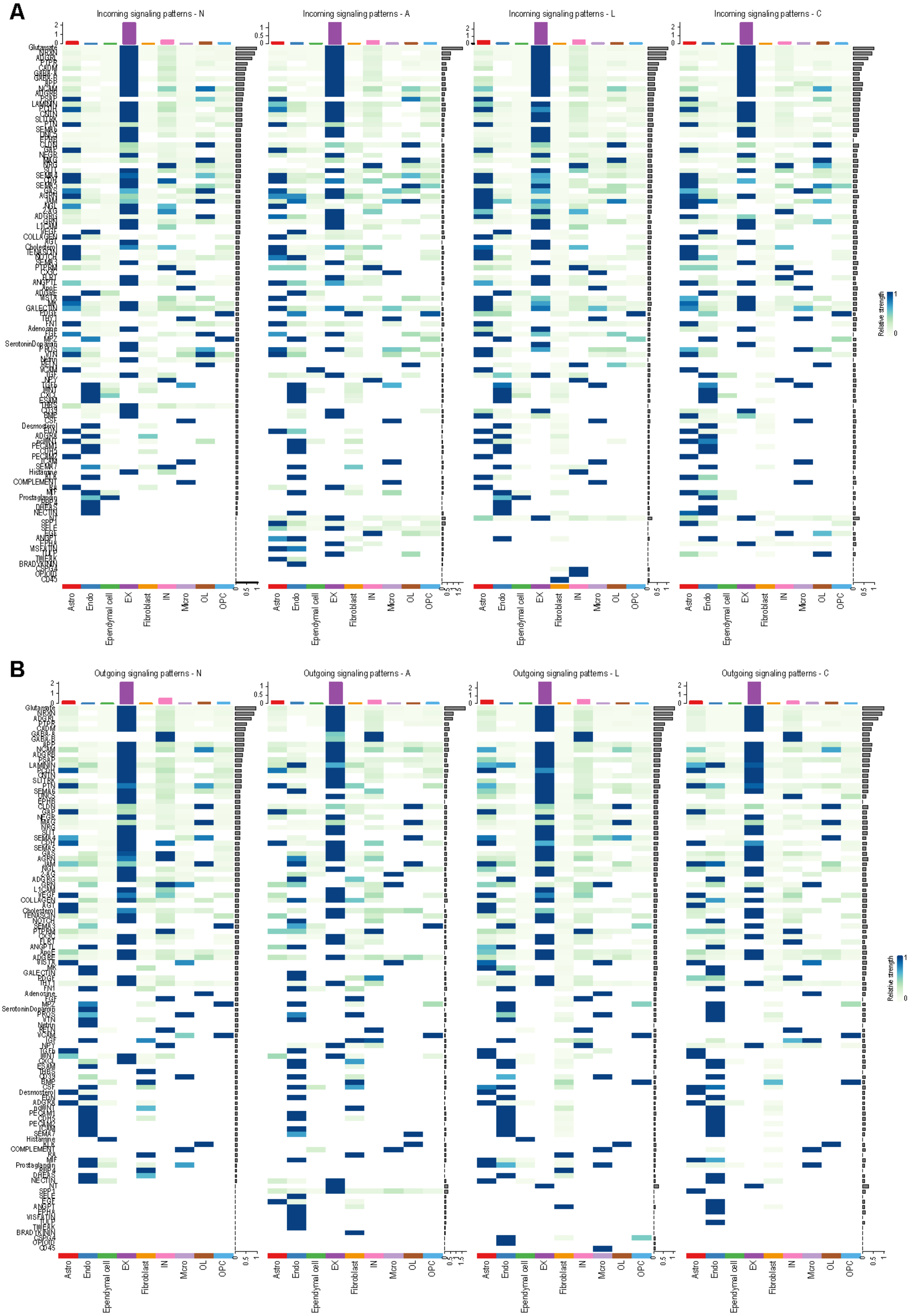
A
Incoming signaling patterns - N
Incoming signaling patterns - A
Incoming signaling patterns - L
Incoming signaling patterns - C
Astro
Endo
Ependymal cell
EX
Fibroblast
IN
Micro
OL
OPC
Relative strength
B
Outgoing signaling patterns - N
Outgoing signaling patterns - A
Outgoing signaling patterns - L
Outgoing signaling patterns - C

Fig. S6 Input (**A**) and output (**B**) signaling pathways of nine cell types in the hippocampus during epileptogenesis.

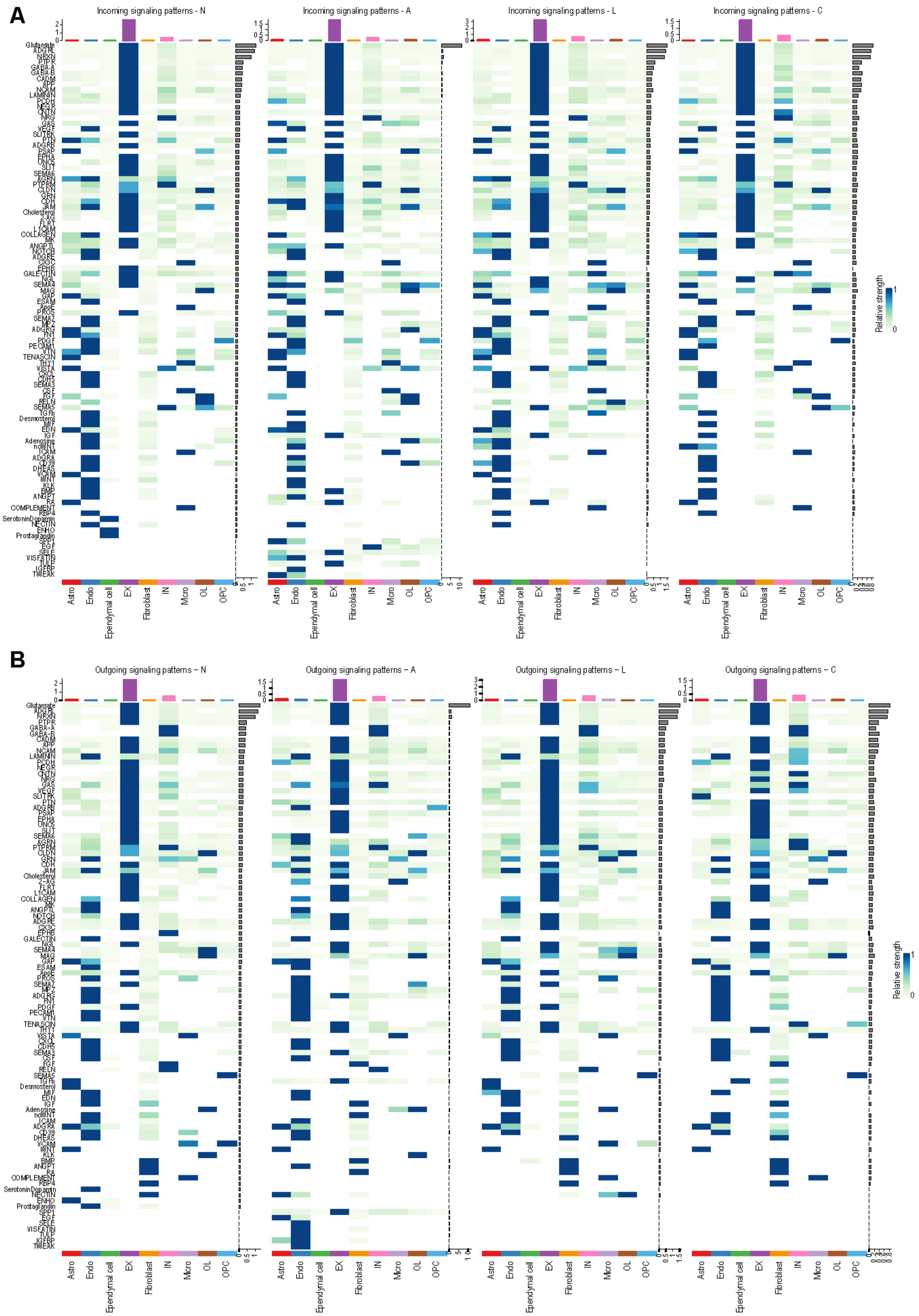
A
Incoming signaling patterns - N
Incoming signaling patterns - A
Incoming signaling patterns - L
Incoming signaling patterns - C
B
Outgoing signaling patterns – N
Outgoing signaling patterns – A
Outgoing signaling patterns – L
Outgoing signaling patterns – C
Astro
Endo
Ependymal cell
EX
Fibroblast
IN
Micro
OL
OPC
Relative strength

Fig. S7 Input (**A**) and output (**B**) signaling pathways of nine cell types in the temporal cortex during epileptogenesis.

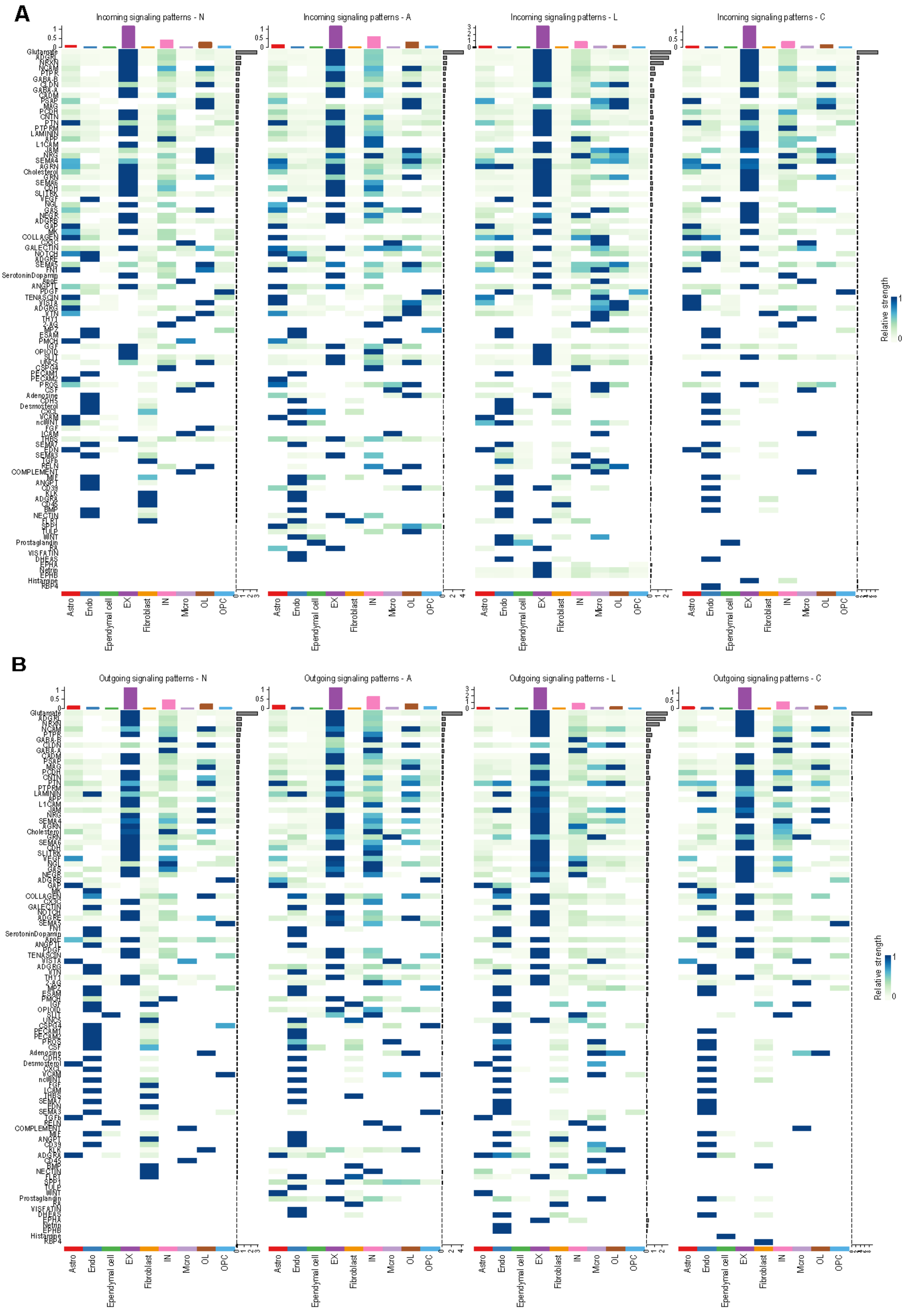
A
Incoming signaling patterns - N
Incoming signaling patterns - A
Incoming signaling patterns - L
Incoming signaling patterns - C
Relative strength
Astro
Endo
Ependymal cell
EX
Fibroblast
IN
Micro
OL
OPC
B
Outgoing signaling patterns - N
Outgoing signaling patterns - A
Outgoing signaling patterns - L
Outgoing signaling patterns - C

Fig. S8 Input (**A**) and output (**B**) signaling pathways of nine cell types in the thalamus during epileptogenesis.

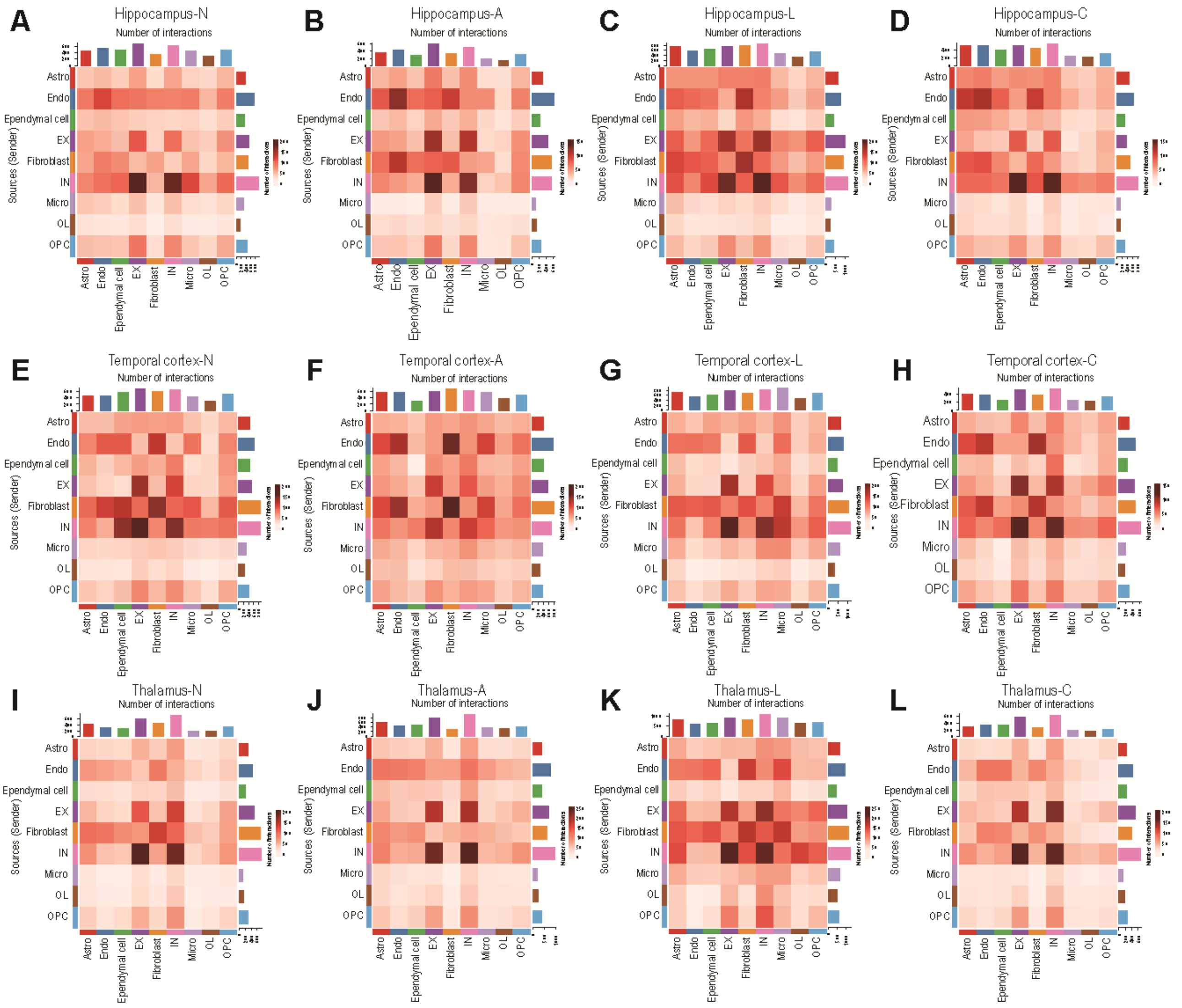

Fig. S9 A-L indicate cell communications among different cell types across the three brain regions during epileptogenesis. **N**, control group; **A**, acute phase; **L**, latent phase; **C**, chronic phase.

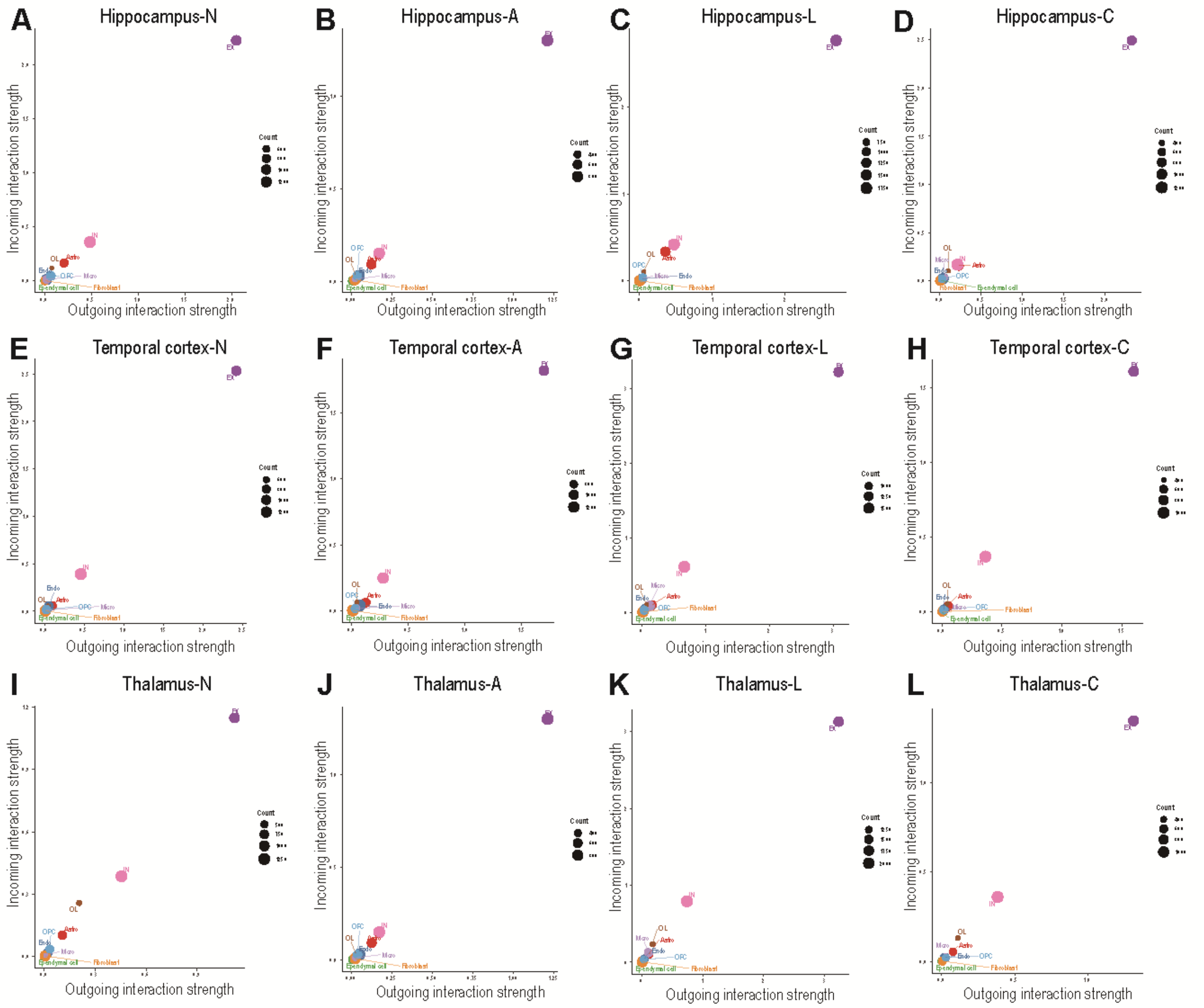

Fig. S10 A-L indicate the dominant senders (sources) and receivers (targets) of cell-cell interactions across different brain regions during epileptogenesis. Excitatory neurons consistently dominate both the input and output of interactive signals. **N**, control group; **A**, acute phase; **L**, latent phase; **C**, chronic phase.

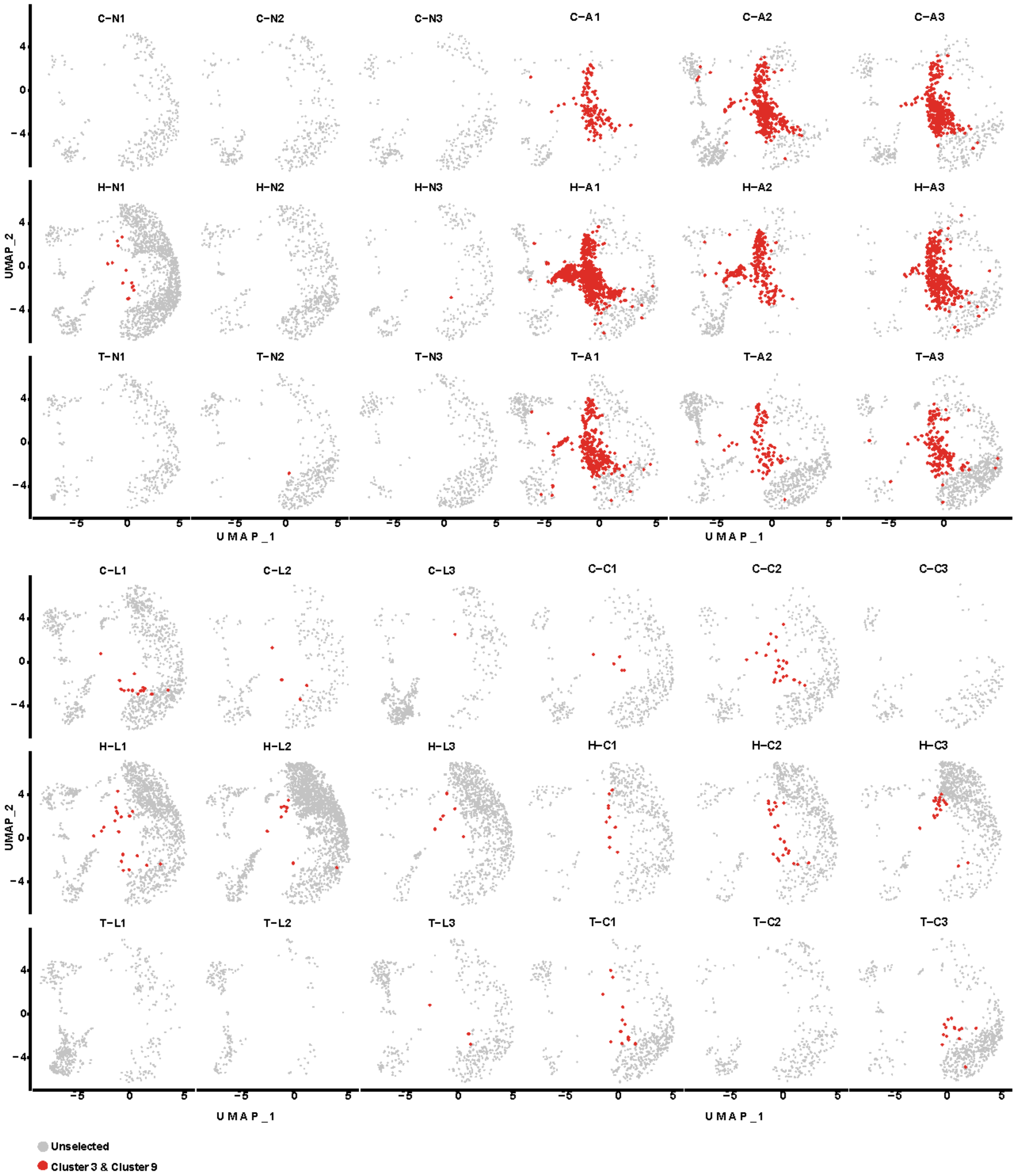

Fig. S11 The distribution of two novel astrocyte clusters (Cluster 3 and Cluster 9) across all individuals and brain regions. These subtypes were identified in the acute phase across all three brain regions and in all three biological replicates: **C** - Temporal cortex, **T** - Thalamus, **H** - Hippocampus. **N**, **A**, **L**, **C**, followed by number, represent different rats in the control, acute, latent, and chronic

phases, respectively.

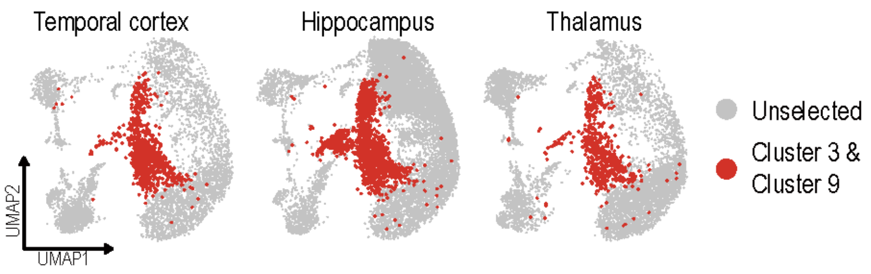

Fig. S12 The distribution of two novel astrocyte clusters (Cluster 3 and Cluster 9) across all brain regions. Compared to the control group, the abundance of genes in Clusters 3 and 9 increased in all three brain regions, with the highest abundance observed in the hippocampus.

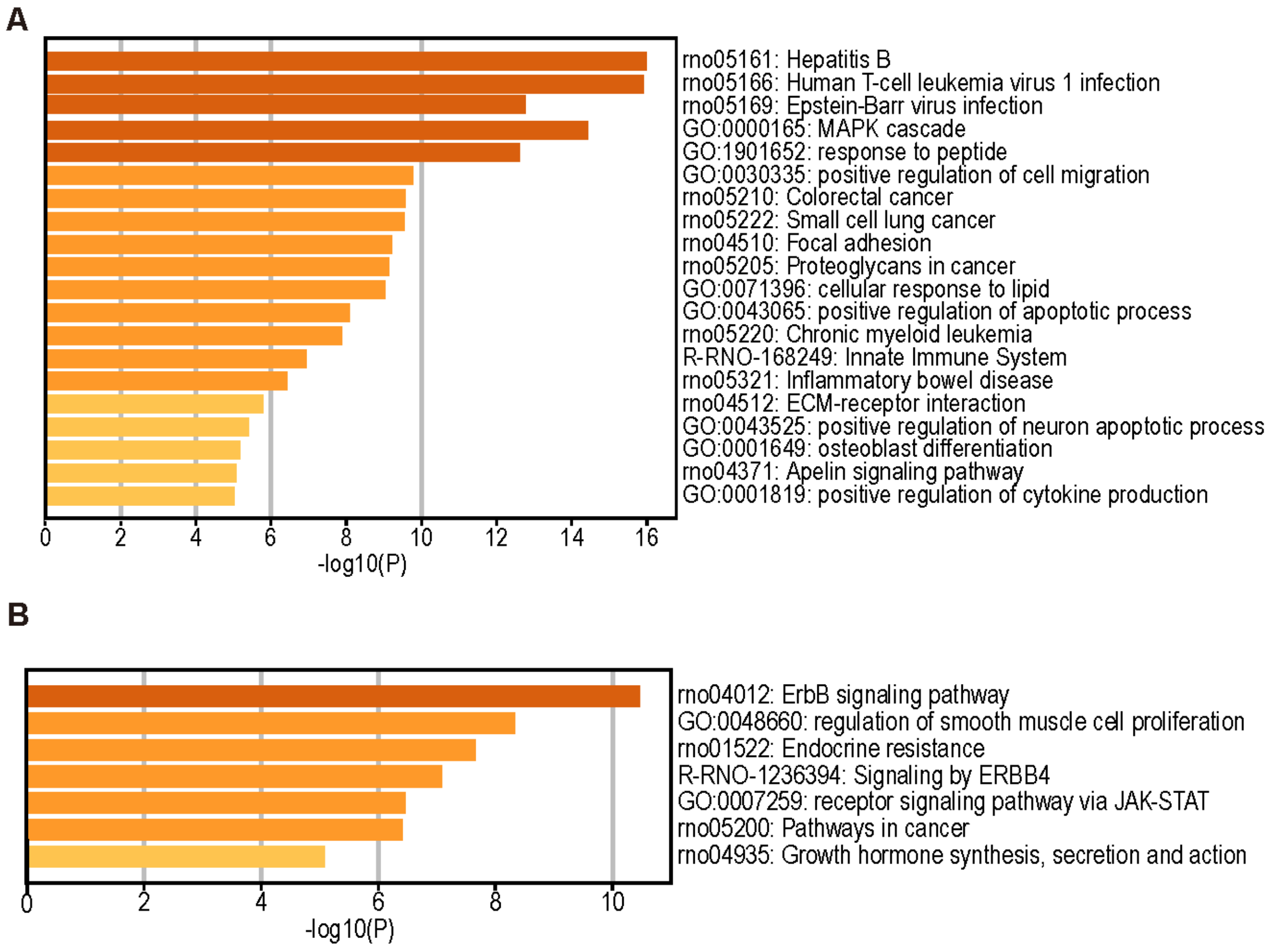

Fig. S13 Gene enrichment analysis of genes involved in the SPP1 pathway (**A**) and the EGF pathway

(**B**).

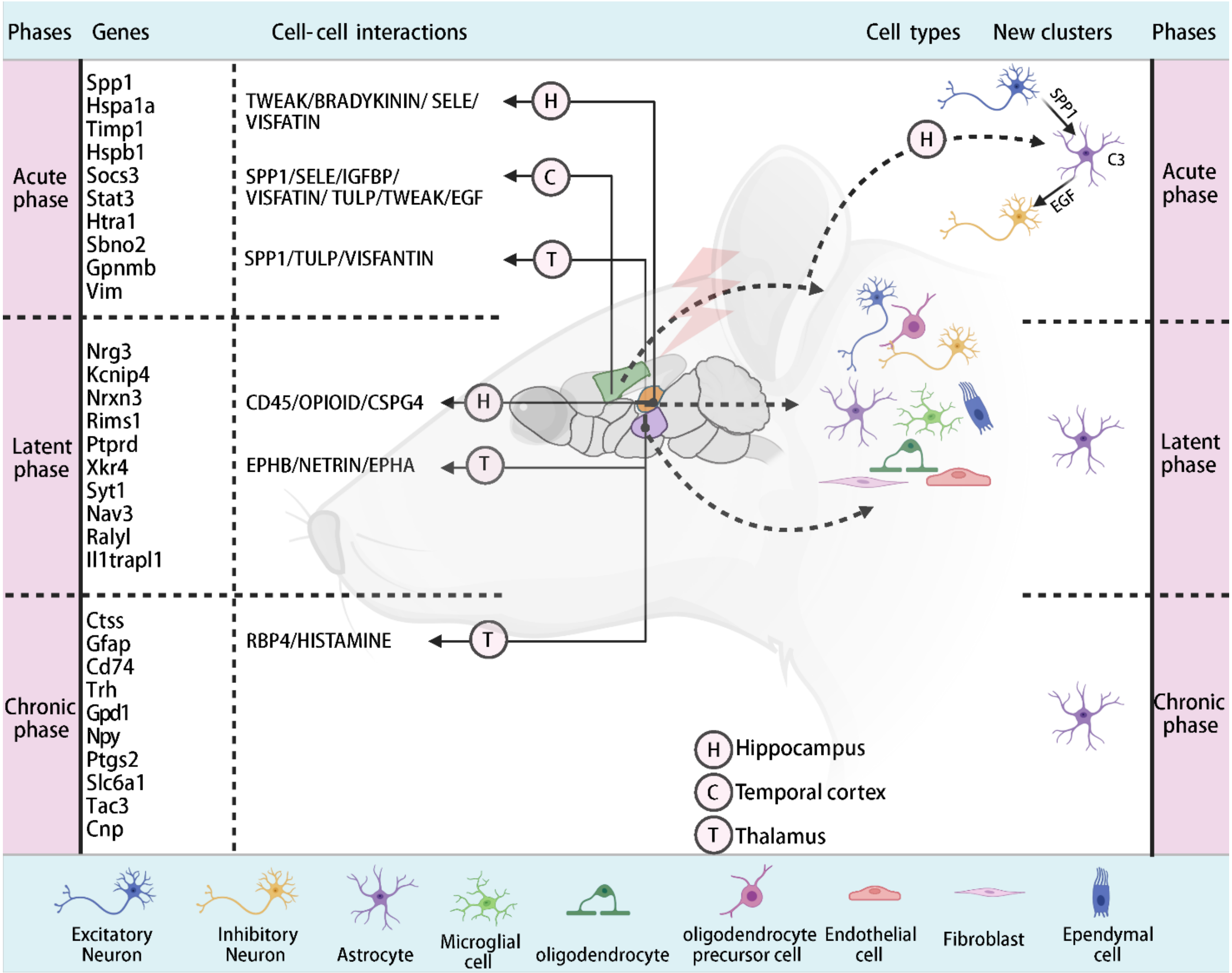

Fig. S14 Dynamic cellular and molecular changes occurring during epileptogenesis. This figure summarizes critical genes, specific cell–cell interaction pathways, and novel astrocyte clusters identified across the three brain regions during different phases of epileptogenesis. The schematic diagram was created using BioRender.com.

Table S1 Detailed information on the latent period and spontaneous recurrent seizures (SRSs) observed in the chronic phase. A total of seven rats were included in the epilepsy group.

Table S2 Comprehensive data on the rats used for snRNA-seq, including the cell types identified for each sample. **N**, control group; **A**, acute phase; **L**, latent phase; **C**, chronic phase. Astro-astrocytes, Micro-microglia, EX-excitatory neuron, IN-inhibitory neuron, OL-oligodendrocyte, OPC-oligodendrocyte precursor cell, Endo-endothelial cell.

Table S3 Data on cell abundance in the temporal cortex, hippocampus, and thalamus during the acute, latent, and chronic phases of epileptogenesis. A Spatial FDR < 0.1 is considered indicative of a significant difference. Notable changes in cell abundance were observed in the temporal cortex during the acute and chronic phases, as well as in the thalamus during the latent phase.

Table S4 Differentially expressed genes (DEGs) identified in various cell types across different brain regions and phases of epileptogenesis. Upregulated genes were defined as those with adjusted p values < 0.05 and $\log_2$fold changes > 0.25, while downregulated genes were defined as those with adjusted p values < 0.05 and $\log_2$fold changes < -0.25. **N**, control group; **A**, acute phase; **L**, latent phase; **C**, chronic phase. **LC**, temporal cortex; **LH**, hippocampus; **LT**, thalamus. Astro-astrocytes, Micro-microglia, EX-excitatory neuron, IN-inhibitory neuron, OL-oligodendrocyte, OPC-oligodendrocyte precursor cell, Endo-endothelial cell.

Table S5 Common genes across different brain regions and phases within each cell type. Gene expression changes are indicated in red for upregulation and in blue for downregulation. Genes highlighted in yellow indicate those that show both upregulation and downregulation across the groups. Astro-astrocytes, Micro-microglia, EX-excitatory neuron, IN-inhibitory neuron, OL-oligodendrocyte, OPC-oligodendrocyte precursor cell, Endo-endothelial cell.

Table S6 Time-trend clustering of gene expression patterns. The Mfuzz (2.64.0) was used to perform time-trend clustering of differentially expressed genes (DEGs) under default parameters. DEGs were categorized into four gene clusters based on their expression changes across the control, acute, latent, and chronic phases. **LC**, temporal cortex; **LH**, hippocampus; **LT**, thalamus.

Table S7 Cell–cell communications among various cell types across different brain regions during distinct phases of epileptogenesis. **N**, control group; **A**, acute phase; **L**, latent phase; **C**, chronic phase. Astro-astrocytes, Micro-microglia, EX-excitatory neuron, IN-inhibitory neuron, OL-oligodendrocyte, OPC-oligodendrocyte precursor cell, Endo-endothelial cell.

Table S8 Genes related to the transformation of astrocyte sub-clusters C3 and C9.

Table S9 Cell-cell communications among various cell types, including astrocytes sub-clusters C3 and C9, in the hippocampus during acute phase. Astro-astrocytes, Micro-microglia, EX-excitatory neuron, IN-inhibitory neuron, OL-oligodendrocyte, OPC-oligodendrocyte precursor cell, Endo-endothelial cell.
